# Phosphoproteomics reveals that the hVPS34 regulated SGK3 kinase specifically phosphorylates endosomal proteins including Syntaxin-7, Syntaxin-12, RFIP4 and WDR44

**DOI:** 10.1042/BCJ20190608

**Published:** 2019-10-30

**Authors:** Nazma Malik, Raja S. Nirujogi, Julien Peltier, Thomas Macartney, Melanie Wightman, Alan R. Prescott, Robert Gourlay, Matthias Trost, Dario R. Alessi, Athanasios Karapetsas

**Affiliations:** 1 Medical Research Council (MRC) Protein Phosphorylation and Ubiquitylation Unit, School of Life Sciences, University of Dundee, Dow Street, Dundee DD1 5EH, U.K.; 2 Dundee Imaging Facility, School of Life Sciences, University of Dundee, Dow Street, Dundee DD1 5EH, U.K.

**Keywords:** Akt, phosphoinositide 3-kinase, phosphoproteomics, SGK3, syntaxins

## Abstract

The serum- and glucocorticoid-regulated kinase (SGK) isoforms contribute resistance to cancer therapies targeting the PI3K pathway. SGKs are homologous to Akt and these kinases display overlapping specificity and phosphorylate several substrates at the same residues, such as TSC2 to promote tumor growth by switching on the mTORC1 pathway. The SGK3 isoform is up-regulated in breast cancer cells treated with PI3K or Akt inhibitors and recruited and activated at endosomes, through its phox homology domain binding to PtdIns(3)P. We undertook genetic and pharmacological phosphoproteomic screens to uncover novel SGK3 substrates. We identified 40 potential novel SGK3 substrates, including four endosomal proteins STX7 (Ser126) and STX12 (Ser139), RFIP4 (Ser527) and WDR44 (Ser346) that were efficiently phosphorylated *in vitro* by SGK3 at the sites identified *in vivo,* but poorly by Akt. We demonstrate that these substrates are inefficiently phosphorylated by Akt as they possess an *n* + 1 residue from the phosphorylation site that is unfavorable for Akt phosphorylation. Phos-tag analysis revealed that stimulation of HEK293 cells with IGF1 to activate SGK3, promoted phosphorylation of a significant fraction of endogenous STX7 and STX12, in a manner that was blocked by knock-out of SGK3 or treatment with a pan SGK inhibitor (14H). SGK3 phosphorylation of STX12 enhanced interaction with the VAMP4/VTI1A/STX6 containing the SNARE complex and promoted plasma membrane localization. Our data reveal novel substrates for SGK3 and suggest a mechanism by which STX7 and STX12 SNARE complexes are regulated by SGK3. They reveal new biomarkers for monitoring SGK3 pathway activity.

## Introduction

The phosphoinositide 3-kinase (PI3K) pathway plays a crucial role in various biological processes, including cell growth, proliferation and metabolism [1]. Most types of tumors harbor mutations that hyperactivate the PI3K pathway and a plethora of inhibitors are being evaluated as anticancer agents [2]. Recently, an orally available PI3K alpha specific inhibitor termed Alpelisib has been approved for treatment of PIK3CA-mutated, hormone receptor-positive advanced breast cancer [3]. Akt inhibitors Capivasertib [4] and Ipatasertib [5] are in late-stage clinical trials for breast cancers bearing PI3K or Akt mutations. However, studies to date indicate that despite encouraging initial responses, the majority of tumors display inherent resistance or develop acquired resistance to PI3K/Akt pathway inhibitors through diverse mechanisms [6,7]. One of these resistance pathways involves up-regulation of the family of serum- and glucocorticoid-regulated kinases, (SGKs), which belong to the AGC family of kinases and bear high similarity to Akt, especially in the catalytic domain. There are three isoforms of SGK termed SGK1, SGK2 and SGK3 [8,9]. Breast cancer cell lines carrying mutations in PTEN or PI3K that also express high levels of SGK1 display inherent resistance to PI3K or Akt inhibitors [10]. Moreover, clinical trials with PI3Kα inhibitors reveal that elevation of SGK1 activity contributes to therapy resistance, at least in part by phosphorylating TSC2 and activating the mTORC1 pathway [11]. In breast cancer cell lines that are initially sensitive to PI3K or Akt inhibitors and that express low levels of SGK1, prolonged treatment with PI3K or Akt inhibitors resulted in up-regulation and activation of SGK3 that also substituted for Akt by phosphorylating TSC2 and ultimately activating mTORC1 [12].

Similar to Akt isoforms, the three isoforms of SGK are activated by phosphorylation of their kinase domain T-loop (Thr320 residue in SGK3) by PDK1 (3-phosphoinositide dependent kinase-1) and at a C-terminal hydrophobic motif (Ser486 in SGK3) by mTORC2 (mTOR complex 2) [13–17]. Phosphorylation of the hydrophobic motif by mTORC2 creates a docking site for PDK1 to phosphorylate the T-loop residue resulting in the activation of SGK isoforms [15–18]. SGK1 and SGK2 unlike Akt isoforms do not possess a regulatory phosphoinositide (PtdIns) binding domain and their activity is therefore tightly controlled by the activity of mTORC2 [9]. SGK3 possesses an N-terminal PtdIns(3)P-binding PX (Phox homology) domain [19]. This targets SGK3 to endosomal membranes, where a large pool of PtdIns(3)P is generated by the Class III PI3K family member, known as hVps34 [17,19,20]. Activation of Class I PI3K can also contribute to the pool of PtdIns(3)P at the endosomal membrane through the sequential degradation of PtdIns(3,4,5)P3 via SHIP2 and INPP4A/4B inositol phosphatases [21,22]. Studies undertaken with selective class 1 and class 3 PI3K inhibitors indicate that both PI3K family members contribute to the activation of SGK3 by growth factors [22].

SGK isoforms phosphorylate substrates in a RXRXXS/T motif similar to Akt [13,23,24]. Indeed, Akt and SGK isoforms phosphorylate many substrates, including TSC2 [11,12], Bad [25], NEDD4L [26–28] and FKHRL-1 [29] at the same residues. The most commonly studied *in vivo* readout of SGK isoform activity is NDRG1 (N-Myc downstream-regulated gene 1), which is efficiently phosphorylated at Thr346 by Akt [30], SGK1 [10] as well as SGK3 [12,22]. Only two SGK3 substrates have been reported, namely AIP4 [31] and FLI-1 [32] that were apparently not phosphorylated by SGK1 and SGK2. To our knowledge, these substrates have not been independently confirmed by others and it is not known whether these proteins are phosphorylated by Akt.

Akt has a strong preference for a large hydrophobic residue such as Phe at the *n* + 1 residue [23]. A substrate peptide for SGK1 termed MURRAYtide derived from NDRG1 was elaborated in a previous work that was poorly phosphorylated by Akt [33]. The MURRAYtide peptide possesses a serine residue at the *n* + 1 position, and changing this to a Phe residue markedly improved phosphorylation by Akt [33]. This work suggested that the *n* + 1 position might represent an important determinant of whether a substrate is phosphorylated by Akt. A prediction from this work is that SGK-selective substrates not phosphorylated by Akt possess non-large hydrophobic residues at the *n* + 1 position. However, the Thr346 residue of NDRG1 that is phosphorylated by Akt and SGK isoforms *in vivo* [10] lies within the RSRSHpTS sequence motif and therefore has a Ser residue as the *n* + 1 residue. This demonstrates that at least in the context of a protein, a substrate bearing non-optimal *n* + 1 residues can be phosphorylated by Akt *in vivo*. In this study, we set out to identify whether there are any SGK3-selective substrates that were not phosphorylated by Akt and study the roles that these might play.

## Materials and methods

### Materials

[γ-^32^P]ATP was purchased from PerkinElmer. Triton X-100, EGTA, sodium orthovanadate, sodium glycerophosphate, sodium fluoride, sodium pyrophosphate, 2-mercaptoethanol, sucrose, Tween 20, Triton X-100 Tris–HCl, sodium chloride and magnesium acetate were from Sigma. PMSF was from Melford. GFP-Trap beads were purchased from Chromotek. Tissue culture reagents, Novex 4–12% Bis-Tris gels and NuPAGE LDS sample buffer were from Invitrogen. Polyethylenimine was from Polysciences. Polybrene was from Sigma. Methanol was from VWR Chemicals. Inhibitor GDC-0941 was from Axon Medchem and MK-2206, AZD8055 and GSK2334470 were purchased from Selleckchem. VPS34-IN1 (1-[[2-[(2-chloropyridin-4yl)amino]-4-(cyclopropylmethyl)-[4,5′-bipyrimidin]-2-yl]amino]-2-methyl-propan-2-ol) was synthesized as described in patent WO 2012085815 A1 as described before [17]. The Sanofi compound 14 h was synthesized as described recently [12]. All plasmids used in this study were generated by the MRC-PPU reagents and Services team (https://mrcppureagents.dundee.ac.uk/). All DNA constructs were verified by DNA sequencing, performed by the MRC-PPU DNA Sequencing and Service (http://www.dnaseq.co.uk). All constructs are available on request from the MRC-PPU reagents webpage (http://mrcppureagents.dundee.ac.uk), and the unique identifier (DU) numbers indicated above provide direct links to the cloning and sequence details.

### Antibodies

The following antibodies were raised in sheep, by the MRC-PPU reagents and Services team (https://mrcppureagents.dundee.ac.uk/) at the University of Dundee, and affinity purified against the indicated antigens: anti-phospho-STX12-Ser139 (SA388 third bleed, raised against the peptide C-Ahx-SIARARAGS*RLSAEERQ (residues 131–140 of human STX12- this antibody works much better with enhanced chemiluminescence (ECL) than LI-COR detection, anti-Akt1 (S695B, third bleed; raised against residues 466–480 of human Akt1: RPHFPQFSYSASGTA), anti-NDRG1 (S276B third bleed; raised against full-length human NDRG1) (DU1557) and anti-SGK3 (S037D second bleed; raised against human SGK3 PX domain comprising residues 1–130 of SGK3) (DU2034). Anti-phospho-Akt Ser473 (#9271), anti-phospho-Akt Thr308 (#4056), anti-phospho-NDRG1 Thr346 (#5482), anti-phospho-rpS6 Ser240/244 (#2215), anti-phospho-PRAS40 Thr246 (#2997) and anti-Rab5(#46449) antibodies were purchased from Cell Signaling Technology. Anti-STX6 (10841-1-AP), anti-STX7 (12322-1-AP), anti-STX12 (14259-1-AP), anti-STX16 (11201-1-AP), anti-VAMP4 (10738-1-AP) and anti-VTI1A (12354-1-AP) were purchased from Proteintech. Anti-STX5 (ab217130), anti-GFP(ab13970) and anti-Na ATPase (ab76020) were purchased from Abcam. Anti-GAPDH (sc-32233) was from Santa Cruz Biotechnology. Secondary antibodies coupled to IRDye680LT or IRDye800CW were obtained from Licor Biosciences. Secondary antibodies coupled to HRP (horseradish peroxidase) were purchased from Thermo Scientific. Secondary antibodies coupled to Alexa Fluor dyes were purchased from Life technologies (Invitrogen).

### Cell culture and cell lysis

HEK293 cells were purchased from the American Tissue Culture Collection (ATCC). Cells were cultured DMEM media supplemented with 10% by vol FBS, 2 mM l-glutamine, 100 U/ml penicillin and 0.1 mg/ml streptomycin. Inhibitor treatments were performed as described in figure legends. The cells were lysed on ice in lysis buffer containing 50 mM Tris–HCl (pH 7.5), 1 mM EGTA, 1 mM sodium orthovanadate, 10 mM sodium glycerophosphate, 10 mM sodium pyrophosphate, 50 mM sodium fluoride, 0.27 M sucrose, 1 mM DTT, 1 mM benzamidine and 0.1 mM PMSF. Lysates were clarified by centrifugation at 11 000***g*** for 20 min at 4°C. Protein concentration was estimated by the Bradford assay (Thermo Scientific). Immunoblotting and immunoprecipitation were performed using standard procedures. The signal was detected using a Li-Cor Biosciences Odyssey System and quantified in Image Studio Lite (Li-Cor) or using the ECL Western Blotting Detection Kit (Amersham) on Amersham Hyperfilm ECL films (Amersham).

### Phosphopeptide enrichment and Tandem mass tags labeling

For PS1, SGK3 knock-out HEK 293 (SGK3 knock-out) cells were generated by the Crispr/Cas9 methodology as described earlier. Wild-type and SGK3 knock-out cells were treated as described in figure legends and lysed using a 2% SDS lysis buffer (2% by mass SDS, 250 mM NaCl, 50 mM HEPES pH 8.5, 1 mM benzamidine, 2 mM PMSF, 2 mM sodium orthovanadate, 10 mM sodium β-glycerophosphate, 5 mM sodium pyrophosphate, 50 mM sodium fluoride, supplemented with protease inhibitor cocktail tablets (Roche) and PhosSTOP phosphatase inhibitors (Roche)). Lysates were heated at 95°C for 5 min prior to sonication and clarification at 14 000 rpm for 15 min. Following the determination of protein concentration by the BCA assay, 25 mg protein was subjected to acetone precipitation. The extracted pellet was resolubilized in 6 M urea/50 mM triethylammonium bicarbonate (TEAB) by sonication and protein concentration determined again by the BCA assay. Protein samples were subsequently reduced with 10 mM DTT and incubated at 56°C for 20 min. Following cooling, samples were alkylated with 30 mM iodoacetamide for 30 min in the dark at room temperature prior to reducing the samples again with 5 mM DTT for 10 min at room temperature. Protein lysates were diluted to 1.5 M urea and digested with Lys-C (Wako, Japan) in a 1 : 200 enzyme:protein ratio overnight at room temperature. Protein extracts were diluted further to a 0.75 M urea concentration, and trypsin (Promega, WI, U.S.A.) was added to a final 1 : 200 enzyme:protein ratio for 16 h at 37°C. Digests were acidified by the addition of trifluoroacetic acid to a final concentration of 1% by vol trifluoroacetic acid. Samples were centrifuged at 4000 rpm for 15 min at 4°C, and the undigested precipitate and excess trypsin were discarded, while the supernatant was retained. Samples were subsequently subjected to C18 solid-phase extraction (SPE) (Sep-Pak, Waters, Milford, MA) to remove salts and impurities. Briefly, Sep-Pak cartridges were activated by adding 4 ml of 100% acetonitrile and equilibrated using 0.1% by vol trifluoroacetic acid by (2× 4 ml). The acidified peptide digest was loaded on to the C18 cartridges. Peptides were cleaned with 2× 4 ml of 0.1% by vol trifluoroacetic acid. Peptides were subsequently eluted with 0.5 ml 60% by vol acetonitrile in 0.1% by vol trifluoroacetic acid. Finally, eluted peptides were lyophilized.

For PS2, HEK293 cells were treated with DMSO, 14H and MK2206 as described in figure legends. The cells were lysed in the same lysis buffer that was used in PS1, and 10 mg of protein amount was prepared for the Lys-C and trypsin digestion as described above and the peptides were desalted as described above. Five percent of the eluate was aliquoted for total proteomic analysis in both PS1 and PS2.

### Phosphopeptide enrichment

For phosphopeptide enrichment, titanium oxide (TiO_2_) beads (Titansphere Phos-TiO_2_ Bulk 10 µm #5010–21315, GL Sciences, Japan) were used [34,35] and prepared by washing with 100% acetonitrile. Tryptic peptides were resuspended in 2 M lactic acid/50% by vol acetonitrile (pH 1.5) by water-bath sonication and centrifuged at 14 000 rpm for 15 min at room temperature, leaving a small pellet consisting of salts and impurities. The supernatant was transferred to fresh Eppendorf tubes and TiO_2_ beads added at a 1 : 2 peptide : bead ratio (for PS1, ∼20 mg of the peptide digest with 40 mg of TiO_2_ beads and for PS2, ∼8 mg of the peptide digest with 16 mg of TiO_2_ bead was used). The bead/peptide mixture was incubated on a mixer at 2000 rpm for 1 h at room temperature followed by centrifugation. Beads were washed twice with 500 µl of 2 M lactic acid/50% by vol acetonitrile (pH 1.5) and once with 500 µl of 0.1% by vol trufluoroacetic acid in 50% by vol acetonitrile (pH 2–4). The beads were resuspended in 200 µl of 0.1% by vol trifluoroacetic acid and loaded onto C8 stage-tips (3M Empore, #2214, U.S.A.) to separate the beads from the flow through, which was discarded. Phosphopeptides were eluted from beads with 30 µl of the elution buffer (50 mM potassium dihydrogen phosphate (K_2_HPO_4_)/5% by mass NH_4_OH) into Eppendorf tubes containing 6% by vol trifluoroacetic acid to quench the ammonia solution. The elution step was repeated another two times and the eluate was then subjected to C18 Sep-Pak stage-tip purification. Briefly, four C18 disks (PN: 2215, 3M Empore, U.S.A.) were packed with 18-gauge needle into a 200 µl pipette tips. The stage-tips were activated with 100 µl of 100% acetonitrile and then equilibrated twice by adding 100 µl of solvent-A (0.1% by vol trifluoroacetic acid). Acidified phosphopeptides (100 µl) were loaded twice and washed twice with 100 µl of solvent-A and then the phosphopeptides were eluted by adding 60 µl of solvent-B (50% by vol acetonitrile in 0.1% by vol trifluoroacetic acid). The elution was repeated again, the eluates were vacuum dried and continued with Tandem mass tag (TMT) labeling.

### TMT labeling

**Figure 1.**
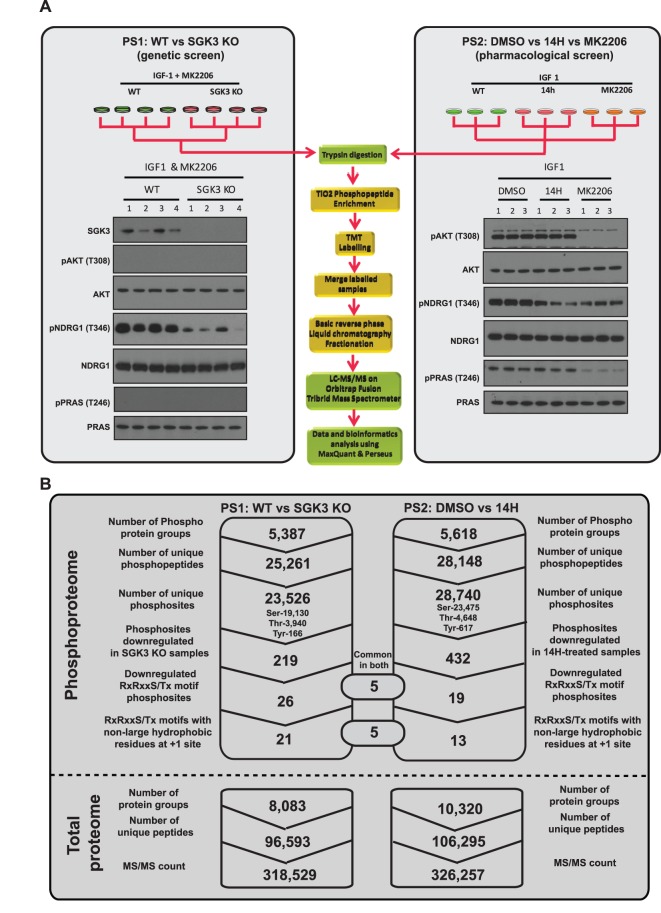
Quantitative phosphoproteomics using TMT labeling to identify selective SGK3 substrates. (**A**) TMT Phosphoproteomics workflow. Wild-type (WT) and SGK3 knock-out (KO) HEK293 cells were serum-starved overnight, treated then with 1 µM MK2206 for 3 h and then stimulated with 50 ng/ml IGF1 for 15 min (PS1, left panel) or WT-HEK293 cells were serum-starved overnight, treated for 1 h with 1 µM MK2206, 3 µM 14H or left untreated (DMSO) and then stimulated with 50 ng/ml IGF1 (PS2, right panel). Cells were lysed in 2% by mass SDS lysis buffer and control immunoblotting was performed with the indicated antibodies. For the TMT labeling, after lysis samples were trypsinized and then phosphopeptide enrichment was performed using TiO_2_ beads after which the peptides were labeled with TMT and mixed (for PS1 ^127^N, ^127^C, ^128^N and ^128^C were labeled to WT-HEK293 samples and ^129^N, ^129^C, ^130^N and ^130^C were labeled to SGK3-KO samples; for PS2 the TMT reporter tags, ^126^N, ^127^N and ^127^C labeled the DMSO treated samples ^128^N, ^128^C and ^129^N labeled the MK2206 treated samples, ^129^C, ^130^N and ^130^C labeled the 14H treated samples). The TMT-labeled samples were subjected to basic-pH reverse liquid chromatography for fractionation and analyzed on Orbitrap Fusion Tribrid Mass spectrometer. (**B**) Summary of the results depicting the identification of unique number of phosphosites and the total number of protein groups from phosphoproteome and total proteome analysis of PS1 and PS2 that were processed through MaxQuant pipeline.

To carry out TMT labeling for both PS1 and PS2 as depicted in Figure 1, the TMT reagents (Thermo Fisher Scientific, TMT 10 plex kit, 0.8 mg Part number:90110) were dissolved in 100 µl anhydrous acetonitrile; phosphopeptide samples were dissolved in 140 µl of 100 mM TEAB buffer. Thirty microliters of anhydrous acetonitrile and 30 µl of the appropriate TMT label (dissolved in acetonitrile) were added to the phosphopeptide samples (140 µl) such that the final acetonitrile concentration was 30% by vol (total volume = 200 µl). For the total proteome, 20 µl anhydrous acetonitrile and 40 µl TMT reagent (dissolved in acetonitrile) were added to the 140 µl peptide sample. The samples were incubated for 2 h at room temperature and vortexed every 30 min after which 10% was aliquoted from each sample to check the labeling efficiency on an Orbitrap Fusion Tribrid mass spectrometer. Furthermore, the reactions were quenched with 5 µl of 5% by vol hydroxylamine and experimental samples were combined in equal amounts and the multiplexed peptide and phosphopeptide samples were lyophilized and subjected to C18 stage-tip desalting as described above.

### Basic-pH reverse-phased fractionation

In the current study, both the TMT-labeled phosphoproteome and total proteome were subjected to basic-pH reversed-phase liquid chromatography (bRPLC) fractionation. Labeled phosphopeptides were solubilized in buffer A (10 mM ammonium formate, pH 10) and separated on an XBridge BEH RPLC column (Waters XBridge BEH C18 Column, 130, 5 µm, 4.6 mm × 250 mm. #186003010) at a flow rate of 0.3 ml/min by applying a non-linear gradient of 7–40% (solvent-B, 90% by vol acetonitrile in 10 mM ammonium formate, pH 10) for 80 min into a total of 96 fractions. The 96 fractions were concatenated into 13 fractions for PS1 and 12 fractions for PS2 in a checkerboard manner and acidified with 30 µl of 1% by vol formic acid (FA). The fractions were subsequently vacuum dried and stored at −80°C until the LC–MS/MS analysis. The total proteome samples from both PS1 and PS2 were also fractionated and subsequently vacuum dried and stored at −80°C deep freezer until the LC–MS/MS analysis.

### LC–MS/MS analysis

Both the PS1 and PS2 phopshoproteome and total proteome samples are analyzed on an Orbitrap Fusion Tribrid Mass spectrometer (Thermo Fisher Scientific, San Jose, U.S.A.) that was in line with the Dionex 3000 RSLC nano-liquid chromatography system, similar to previously published work [36–38]. The peptide samples were loaded on a nano viper trap column (C18, 5 µm, 100 Å, 100 µm × 2 cm; PN: 164562, Thermo Scientific) and resolved on a 50 cm analytical column (2 µm, 100 Å, 75 µm × 50 cm. PN: ES803, Thermo Scientific) and directly electrosprayed using Easy nano LC source (Thermo Fisher Scientific, San Jose, U.S.A.) into the mass spectrometer. Both the TMT-labeled phosphoproteome and total proteome fractions from PS1 and PS2 (phosphoproteome and total proteome) were injected as technical replicates. Phosphopeptide fractions were dissolved in 15 µl of solvent-A buffer (0.1% by vol FA in 3% by vol acetonitrile). The Dionex autosampler loaded the peptides into the trap column at 10 µl/min flow rate using solvent-A (0.1% by vol FA) following the peptide samples were resolved on an analytical column by applying a 3–25% of solvent-B at 300 nl/min flow rate (solvent-B: 80% by vol acetonitrile in 0.1% by vol FA and 3% by vol DMSO) for 180 min and 25–35% solvent-B for 30 min and 35–99% solvent-B for 5 min which was then maintained at 99% solvent-B for 10 min and washed with solvent-A for another 15 min comprising a total run time of 240 min. The mass spectrometer was operated in a data-dependent mode at a top speed comprising 2.5 sec cycle time (MS1 and MS2). The MS1 data were acquired in a scan range of 400–1600 *m*/*z* at 120 000 resolution at 200 *m*/*z* and measured using the ultra high-field Orbitrap mass analyzer. The precursor ions were isolated using quadrupole with an isolation window of 1.6 *m*/*z* which are then fragmented using 37.5% beam-type collisional induced dissociation (HCD) and acquired at 60 000 resolution at 200 *m*/*z* using a ultra high-filed Orbitrap mass analyzer. The AGC and ion injection times for MS1: 3E5 and 50 ms and for MS2: 5E4 and 250 ms were used. The dynamic exclusion was enabled for 35 sec to avoid the repeated sampling of the precursor ions.

### Mass spectrometry data analysis

The mass spectrometry raw data were processed for the protein identification and TMT-based quantification using the MaxQuant pipeline [39,40]. The PS1 total proteome and phosphoproteome were searched using MaxQuant version 1.5.1.7 against the Human Uniprot database (release 2017-02; 42 101sequences). The default common contaminants were selected within the MaxQuant suite. Group-specific parameters: Reporter ion MS2, trypsin as a protease with a maximum of two missed cleavages were allowed. Acetyl protein N-term, deamidation of NQ, oxidation of M and phosphorylation of STY were selected as variable modifications (Phospho STY was excluded for total proteome). Carbamidomethylation of Cys was selected as fixed modification. First search tolerance of 20 ppm and main search tolerance of 5 ppm are allowed. Global parameters: minimum peptide length of seven amino acids and minimum score of 40 and minimum delta score of six for modified peptides were selected. One percent FDR at PSM, peptide and protein level was applied to generate the final output tables. Modified and corresponding unmodified peptides for phosphosites were excluded for accurate protein level quantification. For PS2 both the total proteome and phosphoproteome were processed using MaxQuant version 1.6.0.13 and searched against the Human Uniprot database (release 2017-02; 42 101sequences). The default common contaminants were selected within the MaxQuant suite. Two group-specific parameters were selected. Group 0 for Phosphoproteome and Group 1 for total proteome. Group 0 parameters: Reporter ion MS2 with a minimum reporter PIF (precursor intensity fraction) value of 0.75 was selected to filter out the contaminating peptide fraction for TMT quantification. Acetyl protein N-term, deamidation of NQ, oxidation of M and phosphorylation of STY were selected as variable modifications. Carbamidomethylation of Cys was selected as fixed modification. First search tolerance of 20 ppm and main search tolerance of 5 ppm are allowed. For Group 1, the phosphorylation of STY as a variable modification was excluded. Global parameters: minimum peptide length of seven amino acids and minimum score of 40 and minimum delta score of six for modified peptides were selected. One percent FDR at PSM, peptide and protein level was applied to generate the final output tables. Modified and corresponding unmodified peptides for phosphosites were excluded for accurate protein level quantification. The MaxQuant output tables, Phospho STY sites table and the protein tables were processed separately using the Perseus software [41] suite. Common contaminants and reverse hits were filtered. The TMT quant intensities were log_2_ transformed. The TMT triplicates’ channels were categorized based on the sample conditions (PS1: wild-type and knock-out) and (PS2: DMSO, 1 µM MK2206 and 3 µM 14H). Unquantified proteins were filtered Two-tailed independent *t*-test by applying 5% permutation-based FDR correction was applied to identify the differentially regulated and statistically significant phosphosites and proteins. Phosphosites with *P* < 0.05 and a fold change of 1.5-fold were considered as significantly altered in between the samples.

### Data availability

The mass spectrometry raw data and MaxQuant output tables from both the screens can be accessed using the PRIDE repository identifier PXD014561. (https://www.ebi.ac.uk/pride/archive/). Reviewer login details: Username: reviewer06599@ebi.ac.uk. Password: bwyJsZSf

### Generation of CRISPR–CAS9 knock-out cell lines

The SGK3 HEK293 knock-out cell line has been described previously [22]. To generate STX7 and STX12 HEK293 knock-out cell lines, a modified Cas9 nickase system and the following constructs were used (available at https://mrcppureagents.dundee.ac.uk): DU60563/DU60564 (STX7) and DU60130/DU60132 (STX12). The sgRNA pairs were identified with a low combined off-targeting score [STX7 knock-out-sgRNA1: GACCCCGCCCAGTTGGCCCAG (DU60563); sgRNA2: GAACTCCTGGAGTGTAAGACA (DU60564), STX12 knock-out-sgRNA1: GCCCTCGGGGCCCCAGCTCC (DU60130); sgRNA2: GGGTTCCGGTACATGTCTAA (DU60132)] using the Sanger Centre CRISPR finder tool (http://www.sanger.ac.uk/htgt/wge/find_crisprs). For STX7 knock-out, the guide RNAs target exon 2 of the *STX7* gene, while for STX12 knock-out the guide RNAs target exon 1. The antisense guides (sgRNA2) were cloned onto the spCas9 D10A-expressing pX335 vector (Addgene plasmid no. 42335) and the sense guides (sgRNA1) into the puromycin-selectable pBABED P U6 plasmid (Dundee-modified version of the original Cell Biolabs pBABE plasmid, DU48788). Cells at ∼80% confluency were co-transfected into 10-cm dishes with 2 µg each of the sgRNA plasmid pairs and 20 µl of PEI stock solution (1 mg/ml). Twenty-four hours post transfection, the medium was replaced with fresh one containing 2 µg/ml puromycin in order to select for the cells that have been transfected. After 48 h of selection, the medium was replaced with normal medium and the cells were left to recover for 24 h before performing single-cell sorting using an Influx cell sorter (Becton Dickinson). Single cells were inoculated in individual wells of a 96-well plate containing DMEM supplemented with 20% by vol FBS, 2 mM l-glutamine, 100 units/ml penicillin and 100 mg/ml streptomycin. At ∼80% confluency individual clones were transferred into six-well plates and screened for STX7 or STX12 knock-out by western blotting.

### Generation of GFP-STX7 WT, GFP-STX7 S126A, GFP-STX12 WT, GFP-STX12 S139A HEK293 stable cell lines

To generate stable HEK293 cell lines requiring lentiviral infection, the following pLVX Hygro GFP constructs carrying the gene of interest were used: DU62665 (GFP-STX7 WT), DU62666 (GFP-STX6 S126A), DU62667 (GFP-STX12 WT) and DU62668 (GFP-STX12 S139A). To generate lentiviral particles, 2 µg parent plasmid (containing the gene of interest), 1.5 µg psPAX2 packaging plasmid and 500 ng pMD2.G envelope plasmid were co-transfected into HEK293 FT cells in 10-cm dishes using Lipofectamine (Invitrogen) according to the manufacturer's instructions. After 12 h of transfection, the media were replaced with fresh and the cells were cultured for 24 h. After 24 h, the media containing the lentiviral particles were collected and filtered through a 0.45 µm filter. The virus was either frozen and stored at −80°C for later use or immediately used to infect cell cultures. An amount of 1–5 ml of the virus was applied directly to either HEK293 knock-out STX7 or HEK293 knock-out STX12 cells in the presence of 10 µg/ml polybrene (Hesse et al. 1978). Twenty-four hours post infection, the medium was replaced with fresh one and after 24 h it was changed to the selection medium containing 100 µg/ml of Hygromycin B in order to select for the transduced cells.

### *In vitro* kinase assays

One microgram of kinase was incubated with 0.5–2 µg of the purified protein substrate in the presence of 0.1 mM [γ^32^P]-ATP and 10 mM magnesium acetate in buffer A (50 mM Tris–HCl pH 7.5, 0.1 mM EGTA) at 30°C with shaking at 1100 rpm for 30 min. Reactions were terminated with the LDS sample buffer and resolved by SDS–PAGE electrophoresis. Proteins were detected with Coomassie staining. The gels were imaged with an EPSON scanner, then sandwiched and secured between two sheets of pre-wet cellophane (Bio-Rad) and subsequently dried in a GelAir dryer for 45–60 min. Dried gels were exposed to Amersham Hyperfilm MP overnight, in an autoradiography cassette and the films were later developed using a Konica auto-developer.

### Phosphosite identification by MS and Edman sequencing

Bacterially purified substrate proteins (1 µg) were phosphorylated using recombinant kinase (0.5 µg) in a buffer containing 50 mM Tris–HCl pH 7.5, 0.1 mM EGTA, 10 mM MgAc, 0.1 mM [γ-^32^P]ATP for 80 min at 30°C per reaction. Five to 10 reactions were carried out per phospho-mapping experiment. Reactions were stopped with the LDS sample buffer resolved by SDS–PAGE electrophoresis. Gels were stained with Coomassie blue to stain protein bands. The bands corresponding to the substrate protein were excised and subsequently, washed first with water, then shrunk with acetonitrile and re-swelled with 50 mM Tris–HCl pH 8.0. Afterwards, samples were reduced with 5 mM DTT in 50 mM Tris–HCl pH 8.0 at 65°C for 20 min and then alkylated with 20 mM iodoacetamide in 50 mM Tris–HCl pH 8.0. The gel pieces were then washed for 10 min first in water, then in 50 mM ammonium bicarbonate and finally in 50 mM ammonium bicarbonate 50% by vol acetonitrile. The gel pieces were shrunk in acetonitrile, re-swollen in 50 mM triethylammonium bicarbonate, and then re-shrunk in acetonitrile. Samples were digested overnight with trypsin (5 µg/ml in 50 mM triethylammonium bicarbonate) at 30°C and the peptides were separated on a reverse-phase HPLC Vydac C18 column (Separations Group) with an on-line radioactivity detector. The column was equilibrated in 0.1% by vol trifluoroacetic acid and developed with a linear acetonitrile gradient at a flow rate of 0.2 ml/min. Fractions (0.1 ml each) were collected and analyzed for ^32^P radioactivity by Cerenkov counting with a tricarb scintillation counter. Isolated phosphopeptide fractions were analyzed by liquid chromatography (LC)–MS/MS using a Thermo U3000 RSLC nano-liquid chromatography system (Thermo Fisher Scientific) coupled to a Thermo LTQ-Orbitrap Velos mass spectrometer (Thermo Fisher Scientific) to determine the primary sequence of the phosphopeptides. Data files were searched using Mascot run on an in-house system against a database containing the appropriate substrate sequences, with a 10 ppm mass accuracy for precursor ions, a 0.6 Da tolerance for fragment ions, and allowing for Phospho (ST), Phospho (Y), Oxidation (M) and Dioxidation (M) as variable modifications. Individual MS/MS spectra were inspected using Xcalibur 2.2 (Thermo FisherScientific), and Proteome Discoverer with phosphoRS 3.1 (Thermo Fisher Scientific) was used to assist with phosphosite assignment. The site of phosphorylation of ^32^P-labeled peptides was determined by solid-phase Edman degradation on a Shimadzu PPSQ33A Sequencer of the peptide coupled to the 80 Sequelon-AA membrane (Applied Biosystems).

### Phos-tag SDS–PAGE and immunoblot analysis

Cell lysates were mixed with 4× SDS–PAGE sample buffer [250 mM Tris–HCl, pH 6.8, 8% by mass SDS, 40% by vol glycerol, 0.02% by mass Bromophenol Blue and 4% by vol 2-mercaptoethanol] and heated at 95°C for 5 min. Samples were supplemented with 10 mM MnCl_2_ before boiling and then centrifuged for 1 min at 14 000 rpm. Phos-tag SDS–PAGE was carried out as described previously [42] with some modifications. Gels for Phos-tag SDS–PAGE consisted of a stacking gel [4% by mass acrylamide, 125 mM Tris–HCl, pH 6.8, 0.1% by mass SDS, 0.2% by vol *N,N,N′,N′* tetramethylethylenediamine (TEMED) and 0.08% by mass ammonium persulfate (APS)] and a separating gel [8% by mass acrylamide, 375 mM Tris–HCl, pH 8.8, 0.1% (by mass SDS, 100 µM Phos-tag acrylamide, 200 µM MnCl_2_, 0.1% by vol TEMED and 0.05% by mass APS]. Ten to forty microgram of samples were loaded and electrophoresed at 70 V for the stacking part (∼2 h) and then 120 V for 150 min with the running buffer [25 mM Tris–HCl, 192 mM glycine and 0.1% by mass SDS]. For immunoblot analysis, gels were washed for 10 min in the transfer buffer [48 mM Tris–HCl, 39 mM glycine and 20% by vol methanol] containing 10 mM EDTA and 0.05% my mass SDS three times, followed by one wash in the transfer buffer containing 0.05% SDS for 10 min. Proteins were electrophoretically transferred onto nitrocellulose membranes (Amersham Protran 0.45 µm NC; GE Healthcare) at 100 V for 180 min on ice in the transfer buffer without SDS/EDTA. Transferred membranes were blocked with 5% by mass non-fat dry milk dissolved in TBS-T [20 mM Tris–HCl, pH 7.5, 150 mM NaCl and 0.1% by vol Tween 20] at room temperature for 45 min. Membranes were then incubated with primary antibodies overnight at 4°C. After washing membranes in TBS-T, membranes were incubated with horseradish peroxidase-labeled secondary antibodies at room temperature for 1 h. After washing membranes in TBS-T, protein bands were detected by exposing Amersham Hyperfilm to the membranes using an ECL solution Amersham ECL Western Blotting Detection Reagents (GE Healthcare).

### Co-immunoprecipitation

Cells were lysed in standard lysis buffer as described above. GFP-STX7 WT, GFP-STX7 S126A, GFP-STX12 WT or GFP-STX12 S139A was immunoprecipitated from 2 mg of lysates using GFP-Trap beads (Chromotek) at 4°C for 4 h under rotation. Subsequently, the beads were washed twice with lysis buffer containing 0.45 M NaCl for 2 min at 4°C under rotation and then once with lysis buffer containing 0.15 M NaCl. Protein complexes were eluted from beads using 2× LDS sample buffer with 2% by vol beta-mercaptoethanol, and the co-immunoprecipitating proteins were detected by immunoblot analysis.

### Immunofluorescence

Wild-type and knock-out STX12 HEK293 cells that stably express wild-type GFP-STX12, GFP-STX12 S139A or GFP were seeded on coverslips (precoated with poly-l-lysine) and after 48 h they were serum-starved overnight. Following treatments described in figure legends, cells were fixed with 4% by vol paraformaldehyde and permeabilized with 1% by vol NP-40. Cells were blocked using 1% bovine serum albumin (BSA) in phosphate-buffered-saline (PBS), then incubated for 1 h with primary antibodies, washed three times in 0.2%by mass BSA in PBS, and incubated for 1 h with secondary antibodies. For visualization of GFP-STX12 WT and GFP-STX12 S139A, the GFP signal was enhanced using a chicken anti-GFP antibody (Abcam) followed by anti-chicken secondary antibody conjugated to Alexa Fluor 488. For localization of endosomal compartments, Rab5 was stained with the anti-Rab5 antibody (CST) and secondary anti-mouse conjugated to Alexa Fluor 594. For localization to the plasma membrane, an anti-Na ATPase (Abcam) and anti-rabbit conjugated to Alexa Fluor 594 were used. After incubation with the secondary antibodies, the coverslips were washed three times with 0.2% BSA in PBS. Coverslips were washed once more in water and mounted on slides using ProLong Gold Antifade (ThermoFisher). The images were collected on an LSM710 laser scanning confocal microscope (Carl Zeiss) using the ×63 Plan-Apochromat objective (NA 1.4), using a pinhole chosen to provide a uniform 0.8 µm optical section thickness in all the fluorescence channels.

Identification of plasma membrane positive structures in cells was performed with Volocity (Quorum Technologies). In brief, the Na ATPase-positive structures (Alexa 594) were found using the Find objects method (Objects >2.5 SD from mean image intensity) while the nuclei were found using the Automatic method (Otsu's method), nuclei were separated by using the separate touching objects step (object size 80 µm^2^) and small structures were excluded (<30 µm^2^). The summed GFP (488 signal) pixel intensities for the plasma membrane objects were normalized to the number of cells in the image by dividing by the number of nuclei in the image. Error bars show SEM for images (10 images per treatment).

### Statistical analysis

All experiments were performed at least in duplicates and at least twice. Each figure legend describes the statistical analysis that was performed in more detail.

## Results

### Phosphoproteomic screens to identify novel SGK3 substrates

To identify physiological substrates of SGK3, we compared the phosphoproteomes of wild-type (*n* = 4) and SGK3 knock-out (*n* = 4) serum-starved HEK293 cells treated with an Akt inhibitor (1 µM MK2206) prior to stimulation with IGF1 (50 ng/ml, 15 min). This phosphoproteome screen was termed PS1 (Figure 1A left panel). Control immunoblotting analysis confirmed that the dual Akt/SGK NDRG1 substrate was phosphorylated to a much greater extent in the wild-type compared with SGK3 knock-out cells (Figure 1A left panel). The residual phosphorylation of NDRG1 in SGK3 knock-out cells is likely due to low levels of SGK1 activity [22]. We also performed a second screen termed PS2, in which we compared the phosphoproteomes of IGF1-stimulated (50 ng/ml, 15 min), serum-starved wild-type HEK293 cells treated with either no inhibitor (DMSO control *n* = 3) with a pan SGK isoform inhibitor (3 µM 14H, 1 h, *n* = 3) or with an Akt inhibitor (1 µM MK2206, 1 h, *n* = 3) (Figure 1A right panel). Control immunoblotting revealed that phosphorylation of NDRG1 was partially reduced in the SGK inhibitor (14H) or Akt inhibitor (MKK2206) treated samples (Figure 1A right panel), similar to what has been observed in a previous study [22]. Phosphorylation of the Akt-selective substrate PRAS40 that is not phosphorylated by SGK isoforms was ablated by the Akt inhibitor (MK2206) but not the SGK inhibitor (14H) treatment (Figure 1A).

Phosphoproteomic analysis was undertaken using a multiplexed quantitative phosphopeptide workflow outlined in Figure 1A, in which tryptic peptides from each sample were labeled with different isotopically labeled isobaric TMTs and then mixed together prior to mass spectrometry analysis [43]. Mass spectrometry data were analyzed using the MaxQuant environment [44,45]. All the mass spectrometry data for these screens have been deposited on the PRIDE database [42] with the accession number PXD014561. For PS1 we quantified >25 000 phosphopeptides from 5387 protein groups and >28 000 phosphopetides from 5618 protein groups in PS2 (Figure 1B). For PS1, 219 phosphorylation sites were significantly reduced in all four replicates of the SGK3 knock-out samples. For PS2, 432 phosphorylation sites were significantly reduced in all three replicates of the SGK inhibitor (14H) treated samples (Figure 1B). The independently acquired total proteome measurements confirmed that the detected phosphorylation changes in PS1 and PS2 were not due to altered protein abundances (changes as determined by MaxQuant were less than 2-fold [Supplementary Fig S1 and Supplementary Excel File S1]).

**Figure 2.**
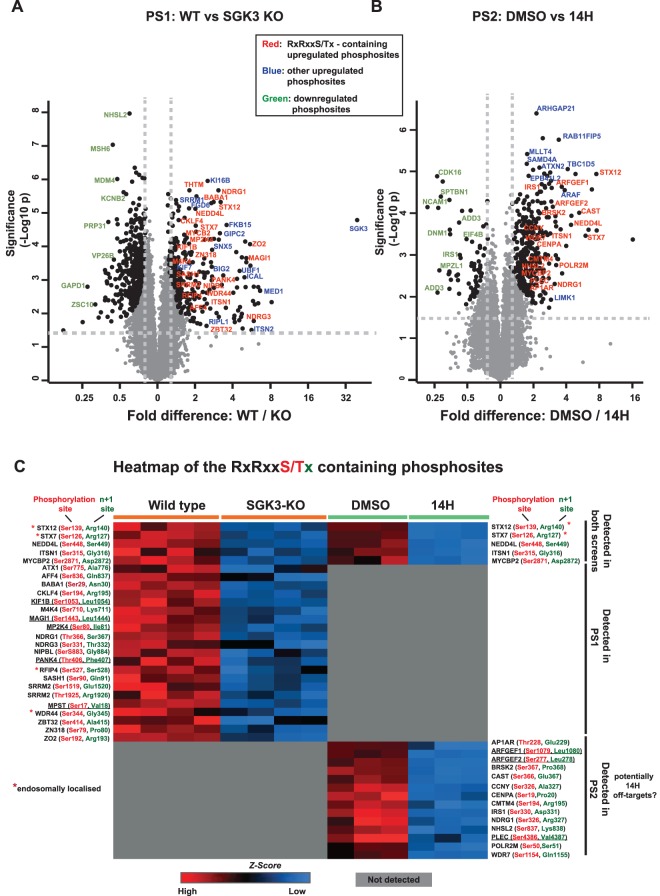
Identification of novel SGK3-selective substrates. Volcano plots depicting the phosphoproteome statistical significance between the HEK293 Wild-type (WT) and SGK3 knock-out (KO) cells (*N* = 4) from PS1 on the left panel (**A**) and for the HEK293 cells treated with DMSO and 14H (*N* = 3) from PS2 on the right panel (**B**). (**A**) For PS1, the significantly enriched phosphosites in WT and KO conditions are highlighted in enlarged black circles along with the color-coded text; In red fonts phosphosites that are specific to RXRXXS/T motif and enriched in the wild-type samples, in blue other phosphosites enriched in the wild-type samples and in green phosphosites enriched in the SGK3-KO samples. (**B**) For PS2, the significantly enriched phosphosites in DMSO and 14H conditions are highlighted in enlarged black circles along with the color-coded text; In red fonts phosphosites that are specific to RXRXXS/T motif and enriched in the DMSO treated samples, in blue other phosphosites enriched in DMSO treated samples and in green phosphosites enriched in 14H samples. For both screens the statistical significance was defined by the independent two-tailed *t*-test, which is corrected by permutation-based FDR of 5% using the Perseus software. (**C**) Heat map depicting the summary of statistically significant RXRXXS/T motif-containing phosphosites identified in the two screens. The log_2_ values of each of the RXRXXS/T phosphosite is *Z*-score normalized and generated the heat map using Perseus software. The phosphorylation site and the +1 site of each motif are highlighted in red and green, respectively. Phosphosites containing a large hydrophobic residue at the +1 site are underlined.

The high confidence phosphosites impacted by SGK3 knock-out in PS1 (Figure 2A) or inhibition of SGK3 in PS2 (Figure 2B) are illustrated by volcano plots. We focused our subsequent analysis on impacted phosphorylation sites lying within the well-characterized Akt and SGK phosphorylation site consensus RXRXXS/T motif [13,23], as these represent good candidates for direct SGK3 substrates. We identified 26 robust phosphorylation sites in PS1 reduced by SGK3 knock-out and 19 robust phosphorylation sites in PS2 inhibited with 14H, within the RXRXXS/T motif (Figs 1B and 2C). Twenty-three of SGK3-dependent sites identified only in PS1 or PS2 were not detected in the other screen, whereas 12 phosphosites in this category were observed not to change in the other screen (Supplementary Excel File S2).

Five of these hits were independently identified in both PS1 and PS2. These included Neural Developmentally Down-Regulated 4-Like E3 ligase (NEDD4L, Ser385) that as discussed in the Introduction is a validated Akt, SGK1 and SGK3 substrates [27,28], and four proteins that to our knowledge are not previously described as SGK or Akt substrates, namely Syntaxin-7 (STX7, Ser126), Syntaxin-12 (STX12, Ser139), Myc-Binding Protein-2 E3 ligase (MYCBP2, Ser2833) and Intersectin-1 (ITSN1, Thr278). The proteomic screens also identified the previously characterized Akt and SGK NDRG1 substrates (Ser366, PS1) and (Ser326, PS2) as well as the related NDRG3 protein (Ser331, PS1). Three other hits corresponded to previously reported Akt substrates, namely BRISC And BRCA1 A Complex Member-1 (BABAM1/ MERIT40) (Ser29, PS1) [46], Ataxin-1 (Ser775, PS1) [47] and WDR44 (Ser344, PS1) [48]. The evidence that WDR44 is phosphorylated by Akt is based on biochemical analysis showing that immunoprecipitated Akt phosphorylated WDR44 at Ser342 and Ser344 [48]. However, mass spectrometry analysis that we have undertaken suggest Ser344 which lies in an RXRXXS/T motif is the likely site of phosphorylation (>95% confidence) (Figure 2C and Supplementary Excel file S3). The Ser342 residue does not lie within an Akt/SGK phosphorylation motif.

As SGK3 is located on the endosome, we perused the list of potential substrates reported to reside on endosomes. This analysis revealed five proteins, namely Intersectin-1 (ITSN1, Ser315, PS1 + PS2, *n* + 1 = Gly) [49], Syntaxin-7 (STX7, Ser126, PS1 + PS2, *n* + 1 = Arg) [50], Syntaxin-12 (STX12, Ser 139, PS1 + PS2, *n* + 1 = Arg) [51], Rab11 family binding protein 4 (RFIP4, Ser527, PS1, *n* + 1 = Ser) [52] and WD Repeat Domain 44 (WDR44, Ser344, PS1, *n* + 1 = Gly) [53].

### STX7, STX12, RFIP4 and WDR44 are phosphorylated efficiently by SGK3 and poorly by Akt *in vitro*

**Figure 3.**
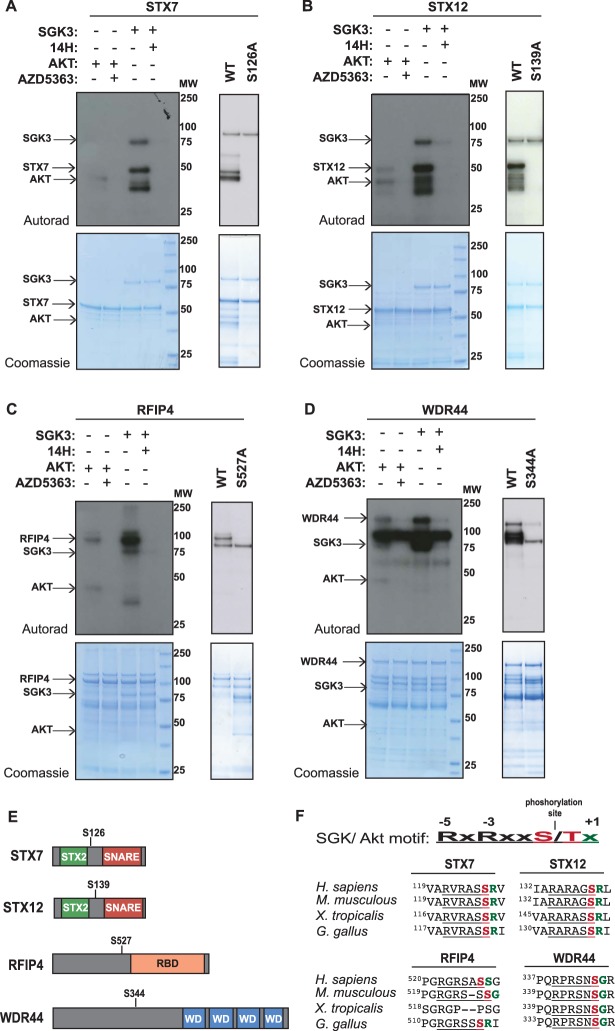
SGK3 but not Akt phosphorylates STX7, STX12, RFIP4 and WDR44 *in vitro*. An amount of 1–2 µg of GST-STX7 (**A**), GST-STX12 (**B**), GST-RFIP4 (**C**) and GST-WDR44 (**D**) were incubated with 0.5 µg active SGK3 or 0.125 µg active Akt and [γ^32^P]-ATP, in the presence or absence of 14H or AZD5363 (1 µM) for 30 min. The reactions were terminated by the addition of SDS loading buffer and separated by SDS–PAGE. Incorporation of [γ^32^P]-ATP was detected by autoradiography (top panels) and proteins were detected by Coomassie staining (bottom panels). Control kinase assays were also performed with the alanine mutants GST-STX7 S126A, GST-STX12 S139A, GST-RFIP4 S527A and GST-WDR44 S344A. (**E**) Schematic representation of STX7, STX12, RFIP4 and WDR44 domain organization. The location of the sites phosphorylated by SGK3 is also indicated. (**F**) Sequence alignment of the motifs phosphorylated by SGK3 in STX7, STX12, RFIP4 and WDR44 in the indicated species. The alignment was performed using the Clustal omega tool (https://www.ebi.ac.uk/Tools/msa/clustalo/).

We next tested whether the potential substrates identified in PS1 and PS2 were phosphorylated by SGK3 and Akt *in vitro* using an *in vitro* ^32^P-γ-MgATP phosphorylation assay to monitor substrate phosphorylation (Figure 3 and Supplementary Fig S2). We were able to express 10 of the hits obtained from PS1 and PS2, namely MPST, PANK4, ITSN1, ATXN1, AFF4, BABA1 STX7, STX12, WDR44 and RFIP4 as full-length proteins in *E. coli*, which included the five endosomal proteins. We employed a concentration of recombinant active SGK3 and Akt1 that phosphorylated NDRG1 to a similar level in a parallel reaction (Supplementary Fig S2A). We observed that five of the substrates tested, namely MPST (*n* + 1 Val Supplementary Fig S2B), ATXN1 (*n* + 1 Ala Supplementary Fig S2D), PANK4 (*n* + 1 Phe Supplementary Fig S2E) and AFF4 (*n* + 1 Gln Supplementary Fig S2F) and one of the endosomal substrates, namely ITSN1 (*n* + 1 Gly Supplementary Fig S2C) were phosphorylated similarly by Akt and SGK3 *in vitro*, in a manner that was blocked by the addition of an Akt (MK2206) or SGK (14H) inhibitor.

In contrast, the other four endosomal localized hits STX7 (*n* + 1 Arg Figure 3A), STX12 (*n* + 1 Arg Figure 3B), RFIP4 (*n* + 1 Gly Figure 3C) and WDR44 (*n* + 1 Ser Figure 3D) were phosphorylated by SGK3 to a significantly greater extent than Akt ranging from 3- to 10-fold higher levels of phosphorylation. Phosphorylation of STX7, STX12, RFIP4 and WDR44 by SGK3 was inhibited by an SGK inhibitor (14H) (Figure 3). Mutation of the site identified in the phosphoproteomic screens (STX7-Ser126, STX12-Ser139, RFIP4-Ser527 and WDR44-Ser344) to Ala markedly inhibited the phosphorylation by SGK3 of all four substrates (Figure 3). We also performed *in vitro* ^32^P-label phospho-mapping analysis that confirmed that SGK3 specifically phosphorylates STX7 on Ser126 and RFIP4 on Ser527 (Supplementary Fig S3). The domain structures of Syntaxin-7, Syntaxin-12, Rab11FIP4 and WDR44 and location of the SGK3 phosphorylation sites are summarized in Figure 3E.

### Mutation of *n* + 1 residue to Phe enhances phosphorylation of STX7, STX12 and RFIP4 by Akt without impacting phosphorylation by SGK3

**Figure 4.**
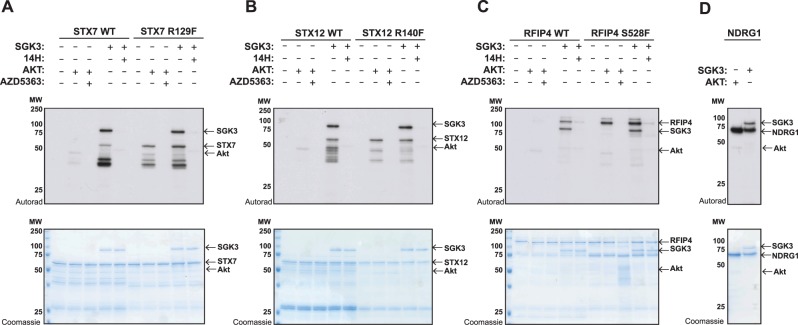
Mutation of *n* + 1 residue to Phe enhances phosphorylation of STX7, STX12 and RFIP4 by Akt. An amount of 1–2 µg of GST-STX7 WT,GST-STX7 R127F (**A**), GST-STX12 WT, GST-STX12 R139F (**B**), GST-RFIP4 WT, GST-RFIP4 S528F (**C**) and GST-NDRG1 (**D**) were incubated with 0.5 µg active SGK3 or 0.1 µg active Akt and [γ^32^P]-ATP, in the presence or absence of 14H or AZD5363 (1 µM) for 30 min. The reactions were terminated by the addition of SDS loading buffer and separated by SDS–PAGE. Incorporation of [γ^32^P]-ATP was detected by autoradiography (top panels) and proteins were detected by Coomassie staining (bottom panels).

As mentioned above STX7, STX12, RFIP4 and WDR44 all possess an unfavorable *n* + 1 residue that might account for why these proteins are poorly phosphorylated by Akt (Figure 3F). To address this question, we mutated the *n* + 1 residue of STX7 (Figure 4A), STX12 (Figure 4B) and RFIP4 (Figure 4C) to a Phe residue that is favorable for phosphorylation by Akt. The recombinant proteins with an *n* + 1 Phe were phosphorylated to a much greater extent than the wild-type proteins by Akt (Figure 4). In contrast, the *n* + 1 Phe mutation did not impact phosphorylation of STX7, STX12 and RFIP4 by SGK3 (Figure 4). Using a concentration of Akt and SGK3 that phosphorylated NDRG1 similarly (Figure 4D), the STX7, STX12 and RFIP4 mutants possessing an *n* + 1 Phe residue were similarly phosphorylated by both Akt and SGK3.

### STX7 and STX12 are efficiently phosphorylated by SGK1 *in vitro*

We next investigated whether STX7, STX12, RFIP4 and WDR44 could be phosphorylated by SGK1 similarly to SGK3 using the *in vitro* ^32^P-γ -MgATP phosphorylation assay (Supplementary Fig S4). STX7 and STX12 were phosphorylated similarly by SGK1 and SGK3 (Supplementary Fig S4A,B). WDR44 (Supplementary Fig S4C) and RFIP4 (Supplementary Fig S4D) were phosphorylated by SGK1 to a lesser extent compared with SGK3. We utilized a concentration of recombinant active SGK1, SGK3 and Akt1 that phosphorylated NDRG1 to similar levels in parallel reactions (Supplementary Fig S4E).

### Endogenous STX7 and STX12 are phosphorylated by SGK3 in HEK293 cells

**Figure 5.**
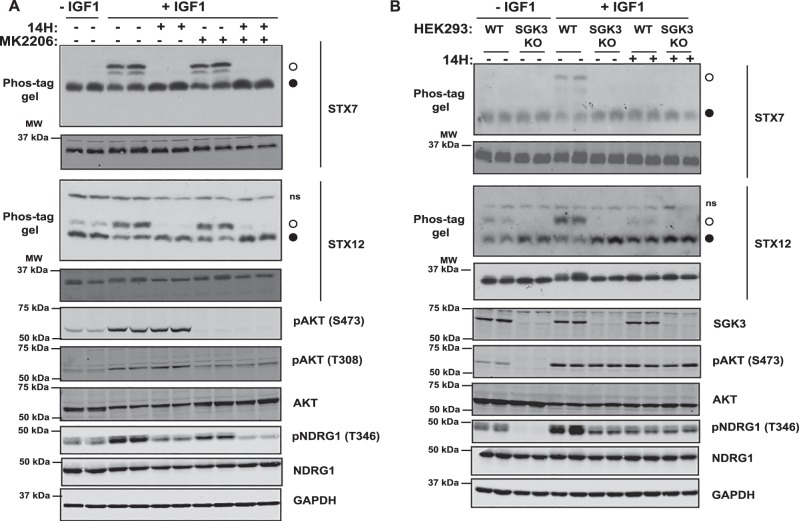
SGK3-dependent phosphorylation of endogenous STX7 and STX12 *in vivo*. (**A**) Wild-type HEK293 cells were serum-starved overnight, then treated for 1 h with 14H (1 µM), MK2206 (1 µM) or with DMSO, followed by stimulation with IGF1 (50 ng/ml) for 15 min. STX7 and STX12 phosphorylation was analyzed by Phos-tag assays (top panels, ° phosphorylated, • non-phosphorylated, ns; non-specific). Control immunoblots were performed on normal gels with the indicated antibodies. Immunoblots were developed using the LI-COR Odyssey CLx Western Blot imaging system analysis with the indicated antibodies at 0.5–1 µg/ml concentration.(**B**) Wild-type (WT) and SGK3 knock-out (KO) HEK293 cells were serum-starved overnight, then treated for 1 h with 14H (1 µM) or with DMSO, followed by stimulation with IGF1 (50 ng/ml) for 15 min. STX7 and STX12 phosphorylation was analyzed by Phos-tag assays (top panels, ° phosphorylated, • non-phosphorylated, ns; non-specific). Control immunoblots were performed on normal gels with the indicated antibodies.

**Figure 6.**
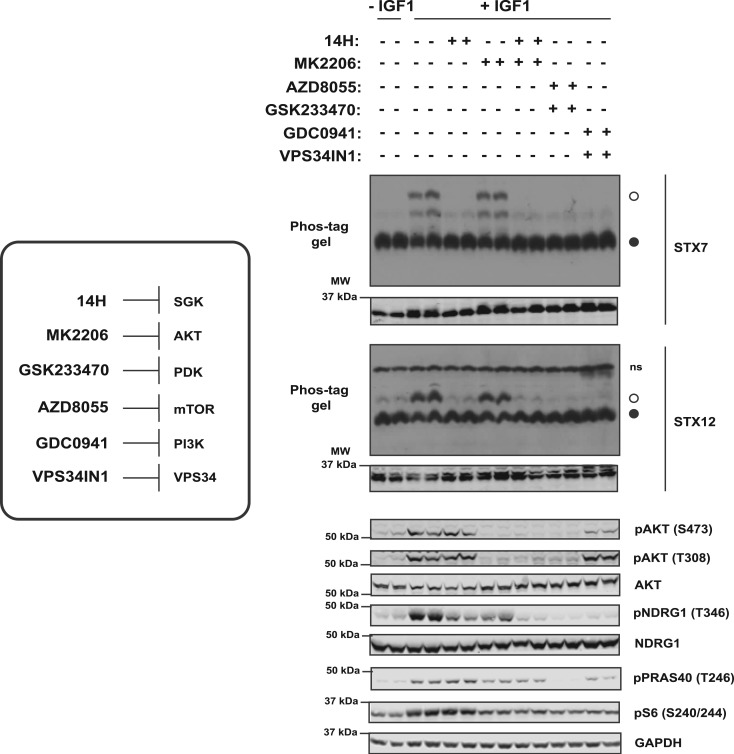
Inhibition of upstream activators of SGK3 blocks phosphorylation of STX7 and STX12 in cells. Wild-type HEK293 cells were serum-starved overnight, then treated for 1 h with 14H (1 µM), MK2206 (1 µM), AZD8055 (1 µM), GSK233470 (1 µM), GDC0941 (1 µM), VPS34-IN1 (1 µM) or with DMSO, followed by stimulation with IGF1 (50 ng/ml) for 15 min. STX7 and STX12 phosphorylation was analyzed by Phos-tag assays (top panels, ° phosphorylated, • non-phosphorylated, ns; non-specific). Control immunoblots were performed on normal gels with the indicated antibodies. Immunoblots were developed using the LI-COR Odyssey CLx Western Blot imaging system analysis with the indicated antibodies at 0.5–1 µg/ml concentration.

We next explored whether the method known as ‘phos-tag gel electrophoresis' that retards the electrophoretic mobility of phosphorylated proteins [54,55] was suitable for analyzing SGK3-mediated phosphorylation of endogenous STX7 and STX12 *in vivo*. We stimulated serum-starved HEK293 cells ± IGF1 (15 min, 50 ng/ml) to activate SGK3 and immunoblotted for total STX7 and STX12 proteins after phos-tag gel electrophoresis. In serum-starved cells STX7 migrated as a single species, but following IGF1 stimulation a striking band-shift was observed indicating that a substantial fraction corresponding to ∼50% of the protein was phosphorylated (Figure 5A). The band-shifted species of STX7 migrated as a major and minor band suggesting that there is phosphorylation at a second minor site. Consistent with phosphorylation being mediated by SGK3, the appearance of both upper bands was blocked with the SGK inhibitor (14H 1 µM), but phosphorylation was not impacted by Akt inhibitor treatment (MK2206, 1 µM) (Figure 5A). Additionally, the IGF1-induced band-shift was blocked in SGK3 knock-out cells (Figure 5B). Control immunoblotting confirmed that IGF1 activated Akt and SGK3 and that the inhibitors and SGK3 knock-out blocked phosphorylation of the expected reporters (Figure 6).

Similarly, IGF1 stimulation (15 min, 50 ng/ml) also induced a major band-shift on a phos-tag gel for STX12 corresponding to ∼60% of the protein becoming phosphorylated (Figure 5A). Phosphorylation of STX12 was blocked following treatment with an SGK inhibitor (14H 1 µM) (Figure 5A) and knock-out of SGK3 (Figure 5B). Only a single shifted phosphorylated species was observed for STX12 that was also present at lower levels in serum-starved cells, indicating basal phosphorylation of this site persists (Figure 5). Consistent with SGK3 mediating the phosphorylation of STX7 and STX12 treatment of cells with kinase inhibitors that suppress the activation of SGK3, namely PDK1 (GSK233470 1 µM [56]), mTOR (AZD8055 1 µM [57]), class 1 PI3K (GDC0941 1 µM [58]) and VPS34 (VPS34-IN1 1 µM [17]) blocked the IGF1-mediated band-shift of STX7 and STX12 (Figure 6). Phylogenetic analysis of the syntaxin family reveals that STX7 and STX12 are closely related and that two other syntaxin proteins possess conserved RxRXXS/T motifs, namely STX5 (Ser9) and STX16 (Ser151) (Supplementary Fig S5A). However, neither of them was identified in our phosphoproteomic screens. Moreover, phos-tag analysis does not reveal detectable phosphorylation of STX5 or STX16 following IGF1 stimulation in HEK293 cells (Supplementary Fig S5B).

### STX7 and STX12 are phosphorylated at Ser126 and Ser139 by SGK3 in HEK293 cells

**Figure 7.**
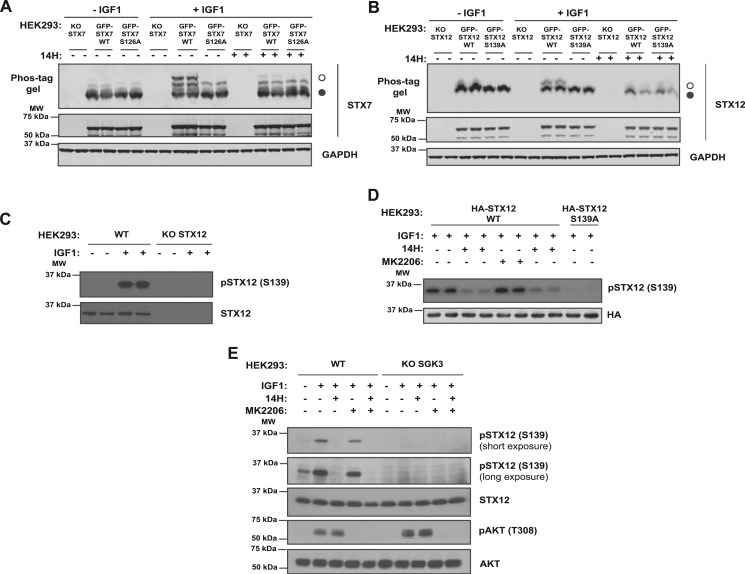
IGF-dependent phosphorylation of STX7 at Ser126 and of STX12 at Ser139 by SGK3. HEK293 stable cells lines expressing (**A**) GFP-STX7 wild type (WT) or the Ala mutant GFP-STX7 S126A and (**B**) GFP-STX12 wild type (WT) or the Ala mutant GFP-STX12 S139A were generated in STX7 knock-out (KO) and STX12 KO HEK293 cells, respectively. (**A**) KO STX7, GFP-STX7 WT and GFP-STX7 S126A HEK293 cells were serum-starved overnight, treated for 1 h with 14H (1 µM) or with DMSO, followed by stimulation with IGF1 (50 ng/ml) for 15 min. STX7 phosphorylation was analyzed by Phos-tag assay (top panels, ° phosphorylated, • non-phosphorylated). Control immunoblots were performed on normal gels with the indicated antibodies. Immunoblots were developed using the LI-COR Odyssey CLx Western Blot imaging system analysis with the indicated antibodies at 0.5–1 µg/ml concentration. (**B**) KO STX12, GFP-STX12 WT and GFP-STX12 S139A HEK293 cells were serum-starved overnight, treated for 1 h with 14H (1 µM) or with DMSO, followed by stimulation with IGF1 (50 ng/ml) for 15 min. STX12 phosphorylation was analyzed by Phos-tag assay (top panels, ° phosphorylated, • non-phosphorylated, ns; non-specific). Control immunoblots were performed on normal gels with the indicated antibodies. Immunoblots were developed using the LI-COR Odyssey CLx Western Blot imaging system analysis with the indicated antibodies at 0.5–1 µg/ml concentration. (**C**) WT or STX12 KO HEK293 were serum-starved overnight, treated with IGF1 (50 ng/ml) for 15 min and samples immunoblotted with the indicated antibodies 0.5–1 µg/ml concentration. The immunoblots were developed using enhanced chemiluminescence and X-ray film. (**D**) HEK293 cells were transfected with plasmids inducing HA-STX12 WT or HA-STX12 S139A. Twenty-four hours post-transfection cells were serum-starved overnight, treated for 1 h with 14H (1 µM), MK2206 (1 µM) or with DMSO, followed by stimulation with IGF1 (50 ng/ml) for 15 min. Samples were analyzed by immunoblot analysis as described in (**D**). (**E**) WT or SGK3 KO HEK293 were serum-starved overnight, treated with IGF1 (50 ng/ml) for 15 min and the samples were analyzed as in (**D**).

To demonstrate that IGF1 was inducing phosphorylation of STX7 at Ser126 and STX12 at Ser139, we generated STX7 and STX12 knock-out HEK293 cells employing CRISPR technology (Supplementary Fig S6) and then re-expressed wild-type or the respective Ala mutants of the SGK3 phosphorylation site (Figure 7). This confirmed that IGF1 failed to induce a major band-shift of the STX7[S126A] (Figure 7A) and STX12[S139A] (Figure 7B) mutants under conditions in which a band-shift was observed for the wild-type proteins. Phosphorylation of the minor species of STX7 was still observed in the STX7[S126A] mutant (Figure 7A), albeit slightly reduced, suggesting that the second phosphorylation site is not greatly impacted by mutation of Ser126. To facilitate analysis of STX12 phosphorylation by SGK3, we generated a sheep polyclonal phospho-specific antibody raised against a phosphopeptide encompassing Ser139 phosphorylated STX12. This antibody detects IGF1-stimulated phosphorylation of endogenous STX12 in wild-type but not CRISPR/CAS9 generated STX12 knock-out HEK293 cells (Figure 7C). Mutation of the Ser139 SGK3 phosphorylation site to Ala or treatment with an SGK3 inhibitor (14H), but not an Akt inhibitor, blocked IGF1-induced STX12 phosphorylation (Figure 7D). CRISPR/CAS9 knock-out of SGK3 also prevented IGF1 from inducing the phosphorylation of STX12 at Ser139 (Figure 7E).

### Evidence that Ser139 phosphorylation of STX12 enhances association with its interacting SNARE complex

**Figure 8.**
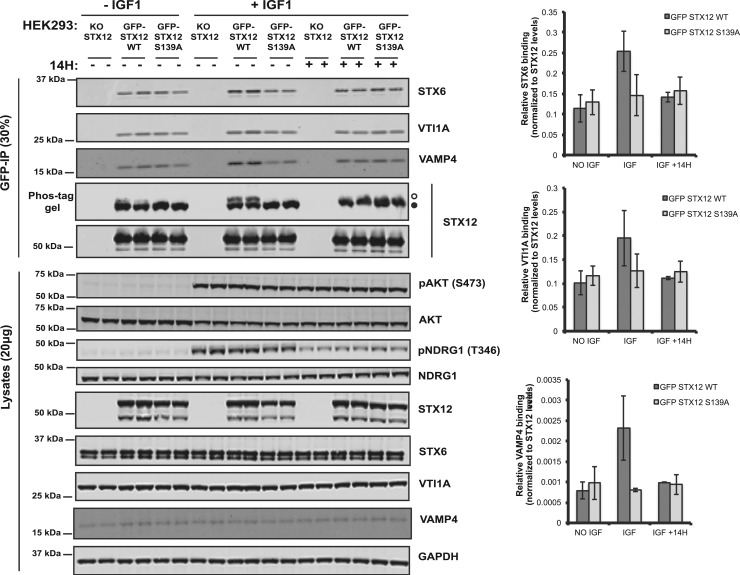
Phosphorylation of STX12 at Ser139 by SGK3 enhances the association with the SNARE complex. (**A**) The indicated knock-out (KO), and KO cells stably re-expressing wild-type or STX[S139A] HEK293 cells were serum-starved overnight, treated for 1 h with 14H (1 µM) or with DMSO, followed by stimulation with IGF1 (50 ng/ml) for 15 min. GFP-STX12 WT and GFP-STX12 S139A were immunoprecipitated using anti-GFP beads and the immunoprecipitates were subjected to western blot analysis using the indicated antibodies. STX12 phosphorylation was analyzed by Phos-tag assay (° phosphorylated, • non-phosphorylated). Control immunoblots were performed on normal gels with the indicated antibodies. Immunoblots were developed using the LI-COR Odyssey CLx Western Blot imaging system analysis with the indicated antibodies at 0.5–1 µg/ml concentration. Quantification of the respective binding is also provided.

Previous work has suggested that STX12 forms a complex with other SNARE proteins including STX6, VTI1a and VAMP4 and this is necessary for the fusion of early endosomes [59,60]. To explore whether phosphorylation of STX12 at Ser139 might impact complex assembly, we immunoprecipitated GFP tagged STX12 wild-type or Ser139Ala mutant that was stably expressed in STX12 knock-out HEK293 cells treated ± IGF1 (50 ng/ml, 15 min) and ±SGK inhibitor (1 µM, 1 h). The immunoprecipitated complexes were analyzed by phos-tag and conventional immunoblot analysis. IGF1 stimulation induced ∼20% phosphorylation of stably expressed wild-type STX12 as judged by phos-tag analysis. STX6, VTI1a and VAMP4 were readily detected in immunoprecipitates derived from GFP-STX12 and GFP-STX12[S139A] cells but not from parental STX12 knock-out cells (that do not express GFP-STX12) (Figure 8). IGF1 stimulation reproducibly increased ∼ 2-fold the amount of STX6, VTI1a and VAMP4 co-immunoprecipitating with wild-type STX12 in three independent experiments. Mutation of the SGK3 Ser139 phosphorylation site to Ala or treatment with the SGK inhibitor (14H) prevented the effect that IGF1 stimulation had on promoting the association of STX12 with STX6, VTI1a and VAMP4 (Figure 8).

### Evidence that Ser139 phosphorylation of STX12 enhances localization to the plasma membrane

**Figure 9.**
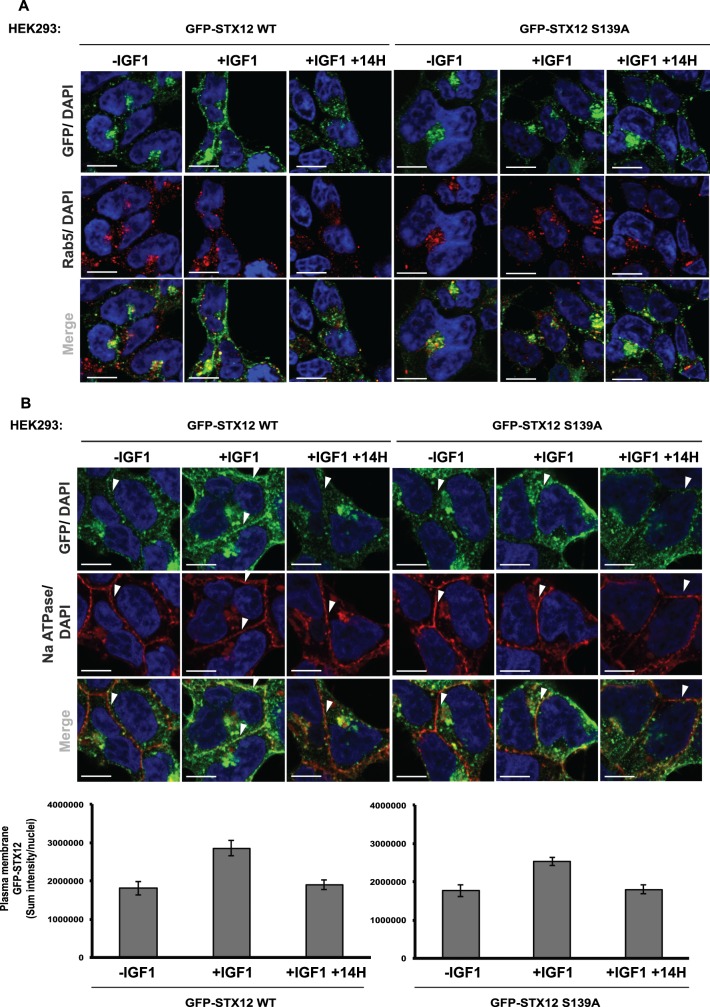
Phosphorylation of STX12 at Ser139 by SGK3 promotes its localization to the plasma membrane. STX12 knock-out (KO) HEK293 cells stably expressing GFP-STX12 WT and GFP-STX12 S139A were seeded on coverslips, serum-starved overnight and treated as indicated with or without 14H (1 µM) for 60 min prior to stimulation with IGF1 for 15 min. Cells were subsequently fixed with 4% by vol paraformaldehyde and GFP distribution was visualized using chicken anti-GFP primary and anti-chicken Alexa Fluor 488 to enhance the GFP signal. (**A**) Co-localization of GFP tagged proteins with an early endosomal Rab5 marker was visualized using mouse anti-Rab5 primary and anti-mouse Alexa Fluor 594 secondary antibody. (**B**) Co-localization to the plasma membrane was visualized using a rabbit anti-Na ATPase and anti-mouse Alexa Fluor 594 secondary antibody. Scale bar; 10 µm. Arrows indicate regions of Na ATPase-positive plasma membrane. Quantification of the GFP-STX12 WT or GFP-STX12 S139A plasma membrane localization is also provided.

To investigate whether phosphorylation of STX12 by SGK3 might affect its cellular localization we performed immunofluorescence analysis of STX12 knock-out HEK293 cells that stably express either wild-type GFP-STX12, the GFP-STX12 Ser139Ala mutant (Figure 9). GFP immunofluorescence of serum-starved cells showed that both the wild-type STX12 and the Ser139Ala mutant were mainly localized in disperse, punctate structures that co-localize with the Rab5 early endosomal marker (Figure 9A). Moreover, in serum-starved cells expressing wild-type GFP-STX12 (Figure 9B), there was a small pool STX12 present at the plasma membrane co-localizing with the Na ATPase plasma membrane marker. However, in serum-starved cells expressing GFP-STX12[S139A] (Figure 9B), less plasma membrane localization of the STX12[S139A] mutant was observed. IGF1 stimulation led to a significant increase in plasma membrane localization of wild-type GFP-STX12 (Figure 9B), which was blocked by treatment with the SGK inhibitor (14H) (Figure 9B). In contrast, IGF1 induced less plasma membrane localization of the GFP-STX12[S139A] mutant compared with the wild type, respectively (Figure 9B).

## Discussion

To our knowledge, this is the first phosphoproteomic study aimed at uncovering physiological substrates for SGK3. In the PS1 phosphoproteomic screen we identified 26 putative substrates phosphorylated at in an RxRXXS/T motif in which phosphorylation was robustly reduced in SGK3 knock-out cells. Two of these substrates were previously reported SGK isoform substrates, namely NEDD4L [26–28] and NDRG1 [30], a third substrate is NDRG3 a homologue of NDRG1 that is also likely be phosphorylated by SGK as one SGK phosphorylation site of NDRG1 is conserved in NDRG3. All 10 other PS1 substrates tested, namely STX7, STX12, ITSN1, ATXN1, AFF4, BAB1, PANK4, RFIP4, MPST and WDR44, were efficiently phosphorylated *in vitro* by SGK3 at the site identified in the PS1 phosphoproteomic screen (Figure 3 and Supplementary Figs. S2, S3). Further work is required to investigate whether the remaining 13 PS1 substrates, namely MYCBP2, CKLF4, M4K4, KIF1B, MAGI, MP2K4, NIPBL, SASH1, SRM2, ZBT32, ZN318 and ZO2 are indeed efficiently phosphorylated by SGK3.

In the PS2 phosphoproteomic screen we identified 19 potential substrates phosphorylated at in an RxRXXS/T motif in which phosphorylation was reduced following treatment with 3 µM of the 14H SGK isoform inhibitor, which inhibits SGK isoforms with an IC50 of 4–10 nM [12]. It should be noted that only five substrates were common in the two phosphoproteomic screens. The 35 other SGK3-dependent phosphorylation sites were only identified in either PS1 or PS2. Twenty-three of the phosphorylation sites in this category were not detected in the other screens, whereas the 12 other phosphosites were observed not to change in the other screens (Supplementary Excel file S2). Further work is required to validate and better characterize these 35 potential SGK3 phosphorylation sites. Although 14H is the most selective and well-characterized SGK3 inhibitor, it is not possible to rule out that impact on substrate phosphorylation observed in PS2 is not a result of an off-target effect of 14H. The selectivity of 14H was assessed using a limited panel of 140 kinases and the key off-target kinases were S6K1 with IC50 of 76 nM and MLK isoforms with IC50 of 100–600 nM [12]. Previous work suggested that 1 µM of 14H only modestly inhibited S6K1 *in vivo* [12]. We were able to demonstrate that three out of the five substrates identified in both PS1 and PS2 were phosphorylated by SGK3, namely STX7, STX12, ITSN1 and all comprise endosomal proteins (Figure 3 and Supplementary Fig. S2). The fourth hit NEDD4L was not tested, but is a well-characterized SGK3 substrate [26–28]). The fifth substrate observed in both PS1 and PS2 was MYCBP2, one of the largest proteins encoded in the human proteome (4678 residues). MYCBP2 encodes an E3 ligase controlling Wallerian degeneration, namely the active process of degeneration that results when a nerve fiber is damaged and the part of the axon distal to the injury degenerates [61]. MycBP2 controls Wallerian degeneration through destabilization of nicotinamide mononucleotide adenyltransferase [62]. It has recently been shown to ubiquitylate substrates on Thr residues rather than Lys [63]. As the same MYCBP2 phosphorylation site (Ser2871) was observed in both PS1 and PS2, MYCBP2 and our data suggest it is a strong candidate for a genuine SGK substrate. Further work is required to establish this and determine whether this phosphorylation is involved in regulating E3 ligase activity and/or Wallerian degeneration. Further work is also required to assess the other 19 substrates identified in the PS2 phosphoproteomic screen that were not observed in PS1, namely AP1AR, ARFGEF1, ARFGEF2, BRSK2, CAST, CCNY, CENPA, CMTM4, IRS1, NDRG1, NHSL2, PLEC, POLR2M and WDR7. From these only NDRG1 is a known as the SGK isoform substrate [30]. A priority for future work would be to rule out that these phosphorylations were not due to off-target effects of 14H and confirm that SGK isoform knock-out or treatment with inhibitors that block SGK isoform activation also suppress phosphorylation of these residues. We did not identify PS1 or PS2 phosphorylation of AIP4 [31] and FLI-1 [32] that were previously reported SGK3-selective substrates.

We focused our analysis exclusively on proteins phosphorylated within RXRXXS/T motifs that are likely to comprise direct SGK3 substrates. The mass spectrometry data set for PS1 and PS2 also contained numerous other phosphorylated substrates not lying in a RXRXXS/T motifs, whose phosphorylation is impacted by SGK3 knock-out or 14H treatment. These data are available for further analysis (Supplementary Excel file) and all data from PS1 and PS2 screens have been deposited on the PRIDE database PXD014561. A motif analysis revealed potentially enriched phosphomotifs such as RXRS (7 statistically significant substrates in PS1 and 2 in PS2) or RXXS/TP (56 statistically significant substrates in PS1 and 230 in PS2) (Supplementary Excel file). Further investigation is needed to understand the mechanism by which these sites might be regulated by SGK3.

It is likely that many of the SGK3 substrates identified in PS1 and PS2 will also comprise Akt substrates. Indeed, five out of 26 substrates identified in PS1 have previously been reported as Akt substrates, namely ATXN1 [47], BABA1/Merit40 [46], NDRG1 [10], NEDD4L [28] and WDR44 [48]. Furthermore, six out of the 10 substrates that were tested, namely NDRG1, MST1, PANK4, ITSN1, ATXN1 and AFF4 were similarly phosphorylated *in vitro* by both Akt and SGK3. Akt has a strong requirement for a large hydrophobic residue at the *n* + 1 position, but SGK isoforms may better tolerate diverse residues at the *n* + 1 position [23,33]. The majority of substrates identified in the PS1 and PS2 screens did not have a large hydrophobic residue at the *n* + 1 position. Only five out of 26 substrates identified in PS1 had a large hydrophobic residue at the *n* + 1 position, namely KF1B-Ser1053(Leu), MAGI-Ser1443 (Leu), MP2K4-Ser80 (Ile) as well as PANK4-Thr406 (Phe) and MPST-Ser17 (Val) that we demonstrated were phosphorylated by both SGK and Akt similarly (Supplementary Fig S2). Three out of 19 substrates in PS2 had an *n* + 1 hydrophobic motif, namely ARFGEF1-Ser1079 (Leu), ARFGEF2-Ser277 (Leu) and PLEC-Ser4386 (Val) that would be predicted to be phosphorylated by both Akt and SGK. The *n* + 1 position of the other substrates identified was Lys/Arg-9 substrates, Ser/Thr-5 substrates, Asp/Glu-4 substrates, Gly/Ala-6 substrates, Asn/Gln-4 and Pro-3 substrates (Figure 2C). Substrates with an *n* + 1 Lys, Arg, Asp and Glu would not be predicted to be well phosphorylated by Akt [23] and are more likely to comprise SGK isoform-selective substrates. A Pro residue at the +1 site is not well tolerated by the AGC kinases [64], thus it is likely that the identified substrates with a Pro at the *n* + 1 position are not directly phosphorylated by the SGK isoforms, but rather phosphorylated by other kinase(s) that favor Pro-directed phosphorylation. A possible candidate for such kinases could include DYRK or CDK isoforms that favor an S/T-P motif. Further work is required to investigate to determine the identity of these Pro-directed kinases and how SGK isoforms might influence their activity.

Recently, it has been reported that Akt can phosphorylate WDR44 at Ser342 and Ser344 and this phosphorylation enhances WDR44 binding to Rab11 and ultimately inhibits ciliogenesis [48]. Our data indicate that Ser344 is the phosphorylation site as mutation of Ser344 to Ala blocked phosphorylation of WDR44 (Figure 3D). We also observed that Akt phosphorylated WDR44 *in vitro* to a much lesser extent than SGK3 and it would thus be interesting to explore whether SGK3-mediated phosphorylation of WDR44 could control Rab11 and ciliogenesis . As phosphorylation of WDR44 by SGK3 and by Akt in previous work [48] has thus far only been studied *in vitro*, it would be important to generate a phospho-specific antibody that recognizes WDR44 phosphorylation at 344 to better study whether it is phosphorylated *in vivo* by Akt and/or SGK isoforms.

**Figure 10.**
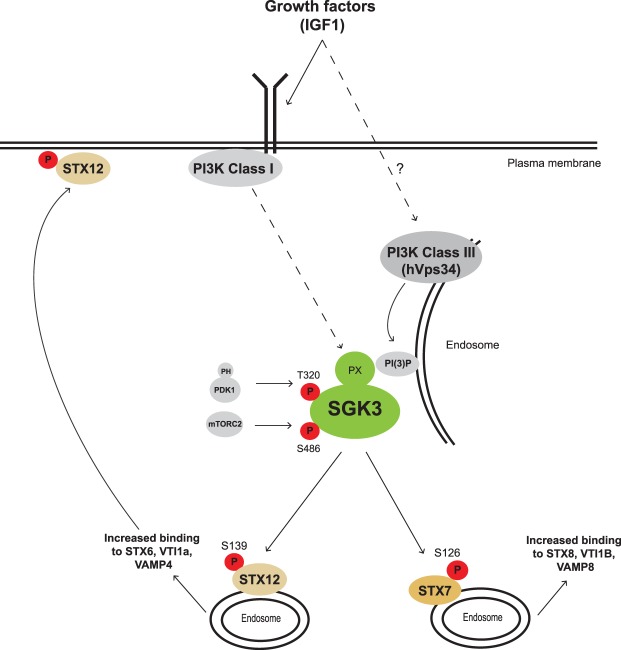
Model of the SGK3 signaling pathway. (**A**) IGF1 stimulates the production of PtdIns(3)P by the Class I and class III Vps34 at the endosomes where SGK3 can be recruited and activated by PDK1 and mTORC2 [22]. Once activated SGK3 phosphorylates its selective substrates STX7 and STX12 that are involved in the endocytic pathway. Phosphorylation of STX12 at Ser 139 by SGK3 that is located just beyond the N-terminal Habc regulatory domain promotes interaction with the t-SNARE proteins syntaxin 6 and Vti1a and the v-SNARE protein VAMP4. Our data suggest that this promotes plasma membrane localization. We propose that assessing phosphorylation of STX7 (Ser126) and STX12 (Ser139) that are specific substrates for SGK2 could be used as key biomarkers for the SGK3 signaling pathway.

The well-characterized NDRG1 substrate that is phosphorylated by both Akt and SGK possesses four phosphorylation sites that have *n* + 1 Ser and Gly residues (Ser330 *n* + 1 = Gly, Thr346 *n* + 1 = Ser, Thr356 *n* + 1 = Ser and Thr366 *n* + 1 = Ser). It is therefore possible that substrates such as RFIP4 and WDR44 that possess a Ser or Gly at the *n* + 1 position could indeed comprise dual Akt or SGK substrates. We have also observed that STX7 and STX12 can be phosphorylated by SGK1 as efficiently as SGK3 *in vitro* (Supplementary Fig S4). As kinase domains of SGK kinases are so similar they are likely to have very similar intrinsic substrate specificity. The HEK293 cells that we have used for our studies express low levels of SGK1 [65] and it is possible that in cell lines that express higher levels of SGK1 or SGK2 that STX7, STX12 or other substrates identified in this study might be phosphorylated by other SGK isoforms. The four SGK3 substrates identified that were poorly phosphorylated by Akt all possessed unfavorable *n* + 1 residues, namely STX7 (*n* + 1 = Arg, STX12 *n* + 1 = Arg), RFIP4 (*n* + 1 = Ser) and WDR44 (*n* + 1 = Gly). Mutation of the residue *n* + 1 residue to Phe in all three of these substrates tested (STX7, STX12 and RFIP4) markedly enhanced phosphorylation by Akt without affecting SGK3 phosphorylation (Figure 4). This provides strong evidence that the *n* + 1 reside comprises a key determinant for Akt versus SGK specificity. Other factors such as cellular co-localization at the endosome and potentially unknown docking and scaffolding interactions are likely to play important roles in mediating the selectivity of phosphorylation. STX7, STX12 and SGK3 all reside at the endosome and it is likely that they also play a major role in accounting for specific phosphorylation of STX7 and STX12 by SGK3 (Figure 10). In future work it would also be interesting to explore further whether ITSN1 that is also endosomally localized is a physiological substrate for SGK3 and whether this can be also phosphorylated by Akt as suggested by our *in vitro* data (Supplementary Fig S2C).

Both STX7 and STX12 belong to the large family of SNARE (soluble *N*-ethylmaleimide (NEM)-sensitive factor attachment receptor) proteins that are involved in membrane fusion in the secretory pathway. All SNARE proteins contain a highly conserved SNARE domain of ∼60 amino acid residues that is responsible for the direct interaction with the SNARE domains of other members of the family [66]. There 15 members of the syntaxin family in mammals (Supplementary Fig S5). They comprise an N-terminal regulatory domain termed Habc (also referred to as the STX2 domain), a SNARE domain and a C-terminal membrane-targeting sequence and with the exception of syntaxin 11, they are all transmembrane proteins. Through their SNARE domain, syntaxins can interact with other SNARE proteins on target membranes, forming a t-SNARE complex, which in turn can bind the SNARE domain of vesicle SNARE proteins (vesicle-associated membrane proteins or VAMPs) located on the cargo-vesicles and thus promote the docking and fusion of the vesicles with the target membranes [67]. Previous work showed that SNARE proteins are highly phosphorylated in the endo-lysosomal system [34,68]. Phylogenetic analysis of the syntaxin family reveals that STX7 and STX12 are closely related and that two other syntaxin proteins possess conserved RxRXXS/T motifs, namely STX5 (Ser9) and STX16 (Ser151) (Supplementary Fig S5). Neither of these sites on STX5 or STX16 was identified in our phosphoproteomic screen. We have undertaken phos-tag analysis of in HEK293 cells and not observed any significant phosphorylation shift of STX5 and STX16 in response to IGF1 stimulation under conditions where STX7 is well phosphorylated (Supplementary Fig S5B).

STX7 is part of a well-characterized SNARE complex comprising the t-SNARE proteins syntaxin 8 and Vti1b and the v-SNARE and VAMP8 and is involved in the fusion of late endosomes with lysosomes [50,69]. Furthermore, it has been reported that CSF-1 stimulation in macrophages induced rapid serine phosphorylation of STX7 and increased its binding to STX8, Vti1b and VAMP8 [70]. It was suggested from bioinformatic and inhibitor analysis that PKC and Akt mediated this CSF-1-induced phosphorylation of Ser-125 and Ser-129, as well as the Ser126 [70], the SGK3 phosphorylation site identified in this study. Our *in vitro* and *in vivo* data suggest that SGK3 is likely to be the kinase that phosphorylates STX7 at Ser 126 as pharmacological inhibition or knocking out of SGK3 prevents STX7 phosphorylation, whereas inhibition of Akt has no effect on STX7 phosphorylation (Figs 5–7). Phos-tag analysis indicates that upon IGF1 stimulation STX7 gets phosphorylated at a second site that we have not mapped, which could comprise the Ser-125 and Ser-129 sites identified previously [70]. We observed that mutation of Ser126 to Ala does not impact phosphorylation of this second site (Figure 7). Our data are consistent with IGF1 inducing phosphorylation of STX7 at Ser126 by SGK3, with at least one other site being phosphorylated by a distinct protein kinase. Additional work is required to identify the other phosphorylation site(s) and determine the identity of the other kinase(s) that phosphorylate STX7.

To our knowledge phosphorylation of STX12 by Akt/SGK isoforms has not been previously studied. STX12 is localized in early and recycling endosomes as well as the plasma membrane, and interacts with the t-SNARE proteins syntaxin 6 and Vti1a and the v-SNARE protein VAMP4 and the formed complex is responsible for the homotypic fusion of early endosomes [59,60]. Furthermore, it has been suggested that STX12 can function at the plasma membrane and can regulate membrane fusion and recycling of plasma membrane proteins [71]. The N-terminal Habc regulatory interacts intramolecularly with the SNARE domain, rendering the protein to a ‘closed conformation' and prohibiting the interaction with the cognate partners and the assembly of the SNARE complex [72–74]. Phosphorylation of various syntaxins can impact their interaction with other SNARE- or regulatory proteins and thus on the formation of the SNARE complex [68,75–77]. For instance, CaMKII can phosphorylate syntaxin-3, at an N-terminal site close to the Habc domain, and this phosphorylation enhances its binding to its SNARE partner, SNAP-25 [78]. Interestingly, the phosphorylation site of STX12 by SGK3 is located at the very end of the N-terminal regulatory domain Habc domain (Figure 3E). Consistent with phosphorylation enhancing SNARE complex formation, the IGF1-stimulated phosphorylation of STX12 at Ser139 by SGK3 increased the binding to the cognate SNARE partners, namely STX6, VTI1a and VAMP4, up to two-fold (Figure 8), whereas no increase was detected with the Ala mutant at this site of STX12 or after treatment with the SGK3 inhibitor 14H. Hence, we propose that SGK3 phosphorylates STX12 at Ser139 at the endosome, leading to a conformational change that renders STX12 in an open-state, facilitating thus its interaction with STX6, VTI1A and VAMP4 and the formation of the SNARE complex (Figure 10). Furthermore, our immunofluorescence data suggest that phosphorylation of STX12 by SGK3 enhances its plasma membrane localization. This is in agreement with previous findings showing that STX12 can bind to a plasma membrane-localized SNARE termed SNAP23 [79,80]. In future work it would be important to explore whether phosphorylated STX12 binds SNAP23.

In summary, through phosphoproteomic screens we have identified the first *in vivo* SGK3-selective substrates, namely STX7 and STX12. According to our model and previous data, upon IGF1 stimulation SGK3 is activated via both PI3K Class I and III pathways and phosphorylates its selective substrates, STX7 and STX12 (Figure 10). Phosphorylation of STX7 [70] and STX12 enhances its interaction with its cognate SNARE partners and the formation of the SNARE complex. At least for STX12, this promotes recruitment to the plasma membrane (Figure 10). Moreover, we identified two other endosomal localized substrates RFIP4 and WDR44 that at least *in vitro* are preferentially phosphorylated by SGK3 and are strong candidates for comprising *in vivo* SGK3 substrates. We also identified numerous other potential new substrates for SGK3 including ITSN1 and MycBP2, which are implicated in diverse biology with links to human disease that would warrant further investigation. Our data suggest monitoring phosphorylation of STX7/STX12 or other SGK3 substrates identified in this study could be exploited as biomarkers of SGK3 activity. Development of such biomarker assays could help identify tumors where SGK3 is a proliferation driver. Such tumors would be predicted to be resistant to Akt pathway suppressors and would instead be better treated by deploying a strategy that targets the SGK pathway.

## Abbreviations

APS: ammonium persulfate
BSA: bovine serum albumin
FA: formic acid
PBS: phosphate-buffered-saline
PI3K: phosphoinositide 3-kinase
SGK: serum- and glucocorticoid-regulated kinase
TEAB: triethylammonium bicarbonate
TEMED: tetramethylethylenediamine
TMT: Tandem mass tag

## Supplementary Material

Supplementary Excel File S1

Supplementary Excel File S2

Supplementary Excel File S3
