# Supplementary material for: Phosphoproteomics reveals that the hVPS34 regulated SGK3 kinase specifically phosphorylates endosomal proteins including Syntaxin-7, Syntaxin-12, RFIP4 and WDR44
Source: Biochem J. 2019 Oct 30;476(20):3081–107. doi: 10.1042/BCJ20190608 (PMC6824681; doi:10.1042/BCJ20190608)
Supplement: Supplementary Figures S1-S6 [file BCJ-476-3081-s4.pdf]

Supplementary Material

Figure S1: Total proteome changes in the phosphoproteomic screens. Volcano plot depicting the total proteome statistical significance between (A) the HEK 293 Wild type (WT) and SGK3 knock-out (KO) (N= 4) from PS1 on the left panel and (B) for the HEK293 cells treated DMSO and 14H conditions (N=3) from PS2 on the right panel. (A) The significantly enriched protein groups in WT HEK293 cells are highlighted in blue filled circles and protein groups that are enriched in KO cells are highlighted in red filled circles. (B) For PS2, the significantly enriched protein groups in DMSO treated cells are highlighted in blue filled circles and protein groups that are enriched in 14H treated cells are highlighted in red filled circles. The statistical significance was defined by the independent two-tailed t-test, which is corrected by permutation-based FDR of 5% using the Perseus software.

Figure S2: MPST1, PANK4, ITSN1, ATXN1 and AFF4 are phosphorylated by both SGK3 and Akt *in vitro*. 1-2 µg of GST-NDRG1 (A), GST-MPST (B), GST-ITSN1 (C), GST-ATXN1 (D), GST-PANK4 (E) and GST-AFF4 (F), were incubated with 0.5 µg active SGK3 or 0.125 µg active Akt and [γ^{32}P]-ATP, in the presence or absence of 14H or AZD5363 (1 µM) for 30 min. The reactions were terminated by the addition of SDS loading buffer and separated by SDS/PAGE. Incorporation of [γ^{32}P]-ATP was detected by autoradiography (top panels) and proteins were detected by Coomassie staining (bottom panels).

Figure S3: Phosphomapping of STX7 and RFIP4 by Edman degradation and mass spectrometry. GST-STX7 (A, B) and GST-RFIP4 (C,D) were incubated with active SGK3 in the presence of [γ^{32}P]-ATP for 80 min. The reactions were terminated by the addition of SDS loading buffer and separated by SDS/PAGE. Proteins were detected by Coomassie staining and the bands corresponding to GST-SXT7 or GST-RFIP4 were excised from the gel and subjected to trypsin digestion. The resultant peptides were separated by HPLC (A, GST-STX7; C, GST-RFIP4) and ^{32}P radioactivity was detected using an online radioactivity detector. The largest peak fractions were analyzed by Edman degradation and mass spectrometry (B, GST-STX7; D, GST-RFIP4).

Figure S4: SGK1 can phosphorylate STX7, STX12, RFIP4 and WDR44 *in vitro*. 1-2 µg of GST-STX7 (A), GST-STX12 (B), GST-WDR44 (C), GST-RFIP4 (D) and GST-NDRG1 (E) were incubated with 0.5 µg active SGK1, 0.25 µg active SGK3 or 0.08 µg active Akt and [γ^{32}P]-ATP, in the presence or absence of 14H or AZD5363 (1 µM) for 30 min. The reactions were terminated by the addition of SDS loading buffer and separated by SDS/PAGE. Incorporation of [γ^{32}P]-ATP was detected by autoradiography (top panels) and proteins were detected by Coomassie staining (bottom panels).

Figure S5: STX5 and STX16 are not phosphorylated by SGK3 in HEK293 cells. (A) Phylogenetic analysis of the Syntaxins**.** Sequence alignment and phylogenetic analysis were performed using Clustal Omega. The syntaxins containing a potential SGK3 phosphomotif are indicated. (B) Wild type (WT) and SGK3 knock-out (KO) HEK293 cells were serum starved overnight, then treated for 1 h with 14H (1 µM), MK2206 (1 µM) or with DMSO, followed by stimulation with IGF1 (50 ng/ml) for 15 min. STX5 and STX16 phosphorylation was analyzed by Phos-tag assays (top panels, top panels, °phosphorylated, •non-phosphorylated). Phos-tag analysis of the same samples for STX7 was performed as a control. Control immunoblots were performed on normal gels with the indicated antibodies. Immunoblots were developed using the LI-COR Odyssey CLx Western Blot imaging system analysis with the indicated antibodies at 0.5-1 µg/mL concentration.

Figure S6: Generation of STX7 and STX12 knock-out **HEK293 cell lines by CRISPR/Cas9**. For generating the STX7 knock-out (KO) cell line the guide RNAs targeted exon 2 of *STX7* gene, while for STX12 KO the guide RNAs targeted exon 1 of *STX12* gene. Two representative KO clones for each cell line are shown. All the clones were blotted for STX7, STX12 and GAPDH as a control.

Figure S1

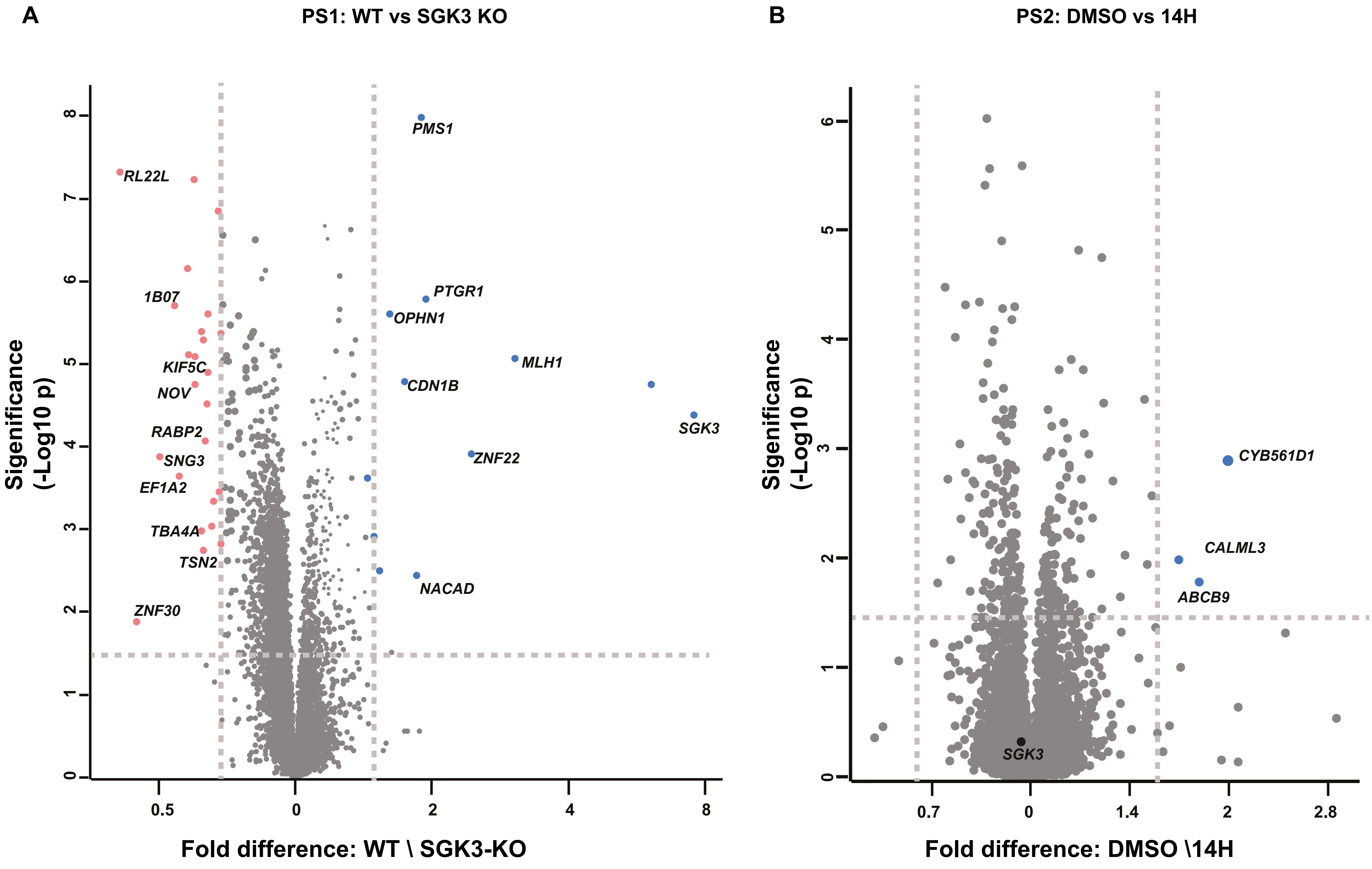
A
PS1: WT vs SGK3 KO
Sigenificance (-Log10 p)
Fold difference: WT \ SGK3-KO
0.5
0
2
4
8
PMS1
RL22L
1B07
PTGR1
OPHN1
MLH1
KIF5C
NOV
CDN1B
SGK3
RABP2
SNG3
ZNF22
EF1A2
TBA4A
TSN2
NACAD
ZNF30
B
PS2: DMSO vs 14H
Sigenificance (-Log10 p)
Fold difference: DMSO \14H
0.7
0
1.4
2
2.8
CYB561D1
CALML3
ABCB9
SGK3

Figure S2

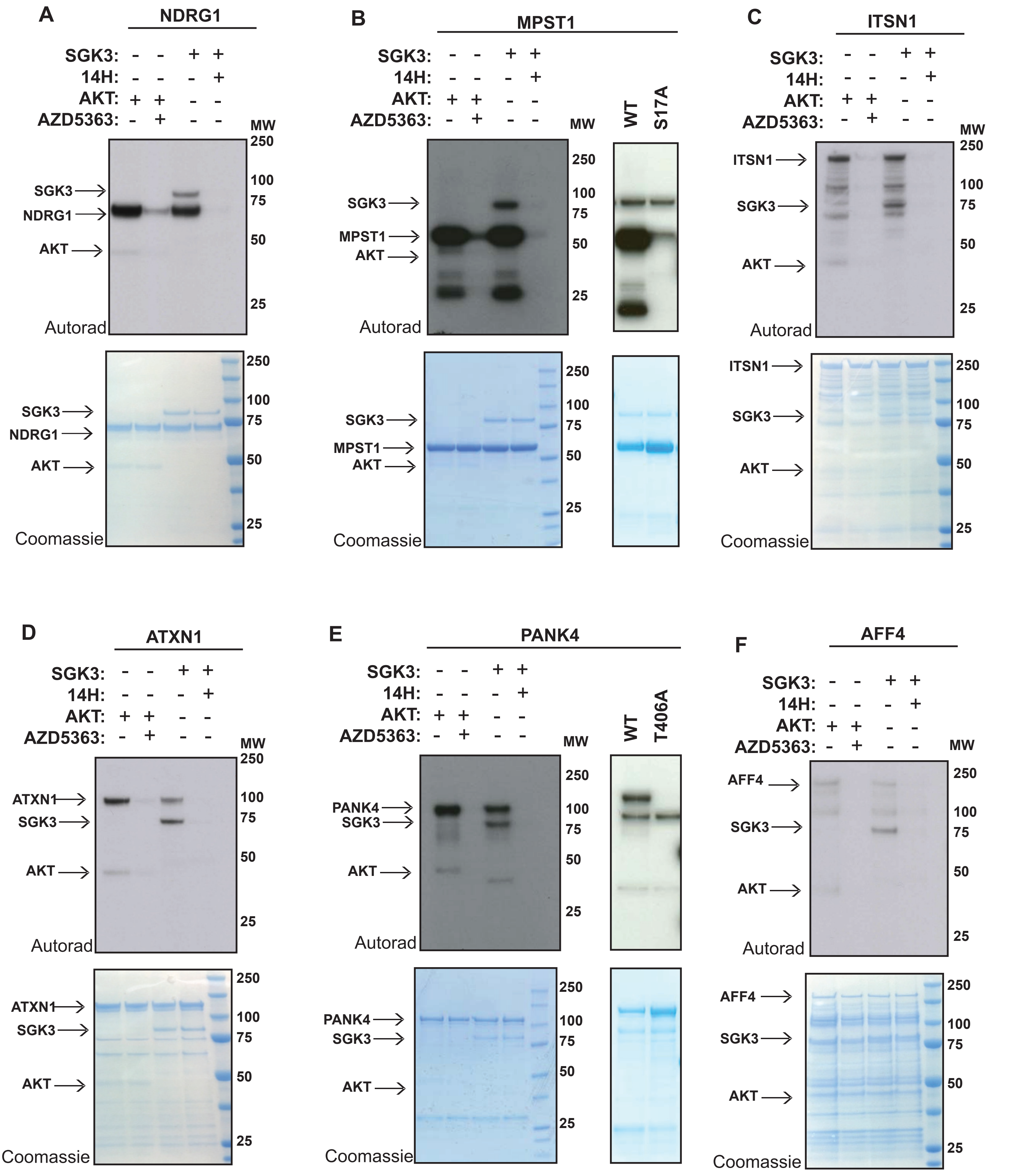

A
NDRG1
SGK3: - - + +
14H: - - - +
AKT: + + - -
AZD5363: - + - -
MW
250
100
75
50
25
SGK3
NDRG1
AKT
Autorad
Coomassie
B
MPST1
SGK3: - - + +
14H: - - - +
AKT: + + - -
AZD5363: - + - -
MW
WT
S17A
SGK3
MPST1
AKT
Autorad
Coomassie
C
ITSN1
SGK3: - - + +
14H: - - - +
AKT: + + - -
AZD5363: - + - -
MW
ITSN1
SGK3
AKT
Autorad
Coomassie
D
ATXN1
SGK3: - - + +
14H: - - - +
AKT: + + - -
AZD5363: - + - -
MW
ATXN1
SGK3
AKT
Autorad
Coomassie
E
PANK4
SGK3: - - + +
14H: - - - +
AKT: + + - -
AZD5363: - + - -
MW
WT
T406A
PANK4
SGK3
AKT
Autorad
Coomassie
F
AFF4
SGK3: - - + +
14H: - - - +
AKT: + + - -
AZD5363: - + - -
MW
AFF4
SGK3
AKT
Autorad
Coomassie

Figure S3

STX7

A

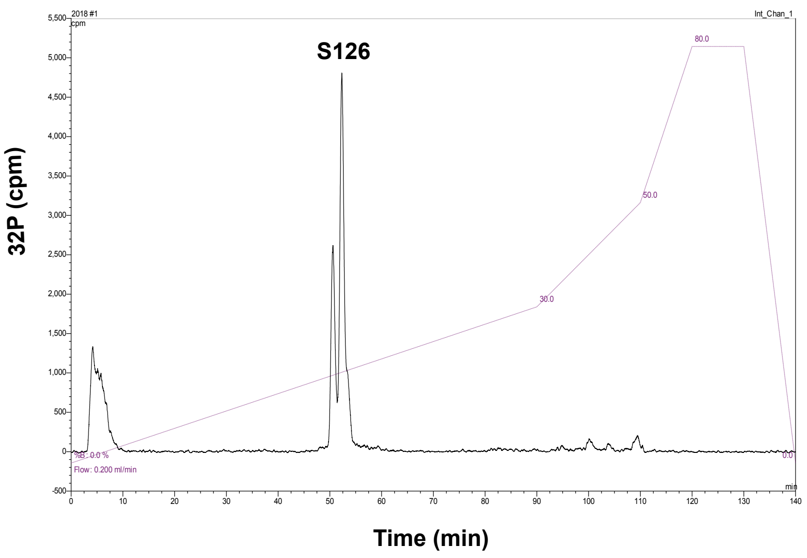

B

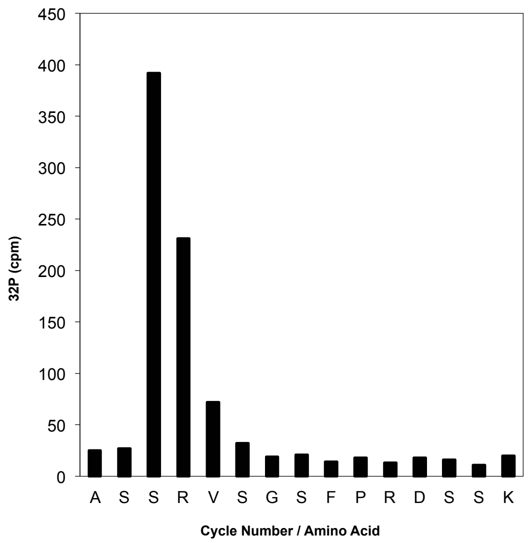

Phosphosite identified as S126
RXRXXS/T
RVRASS

RFIP4

C

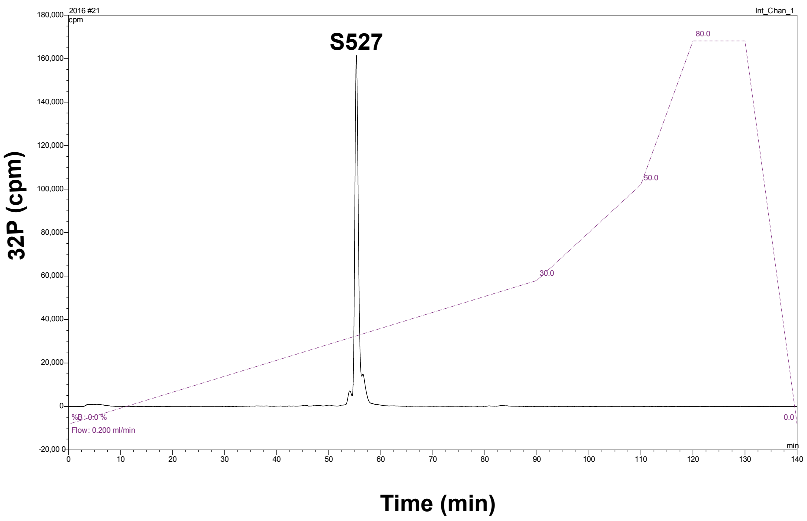

D

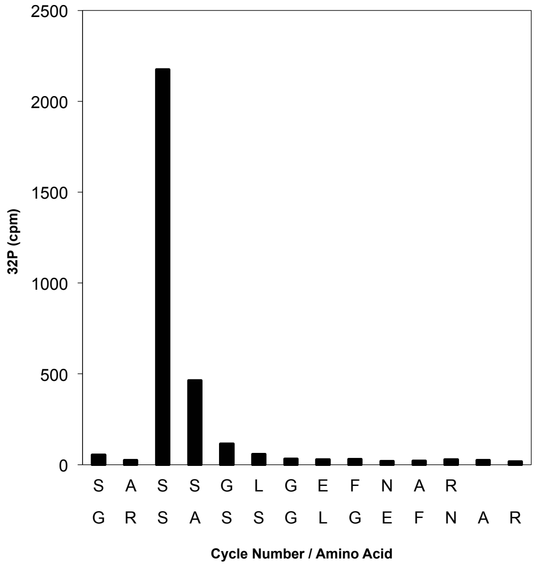

Phosphosite identified as S527
RXRXXS/T
RGRSAS

Figure S4

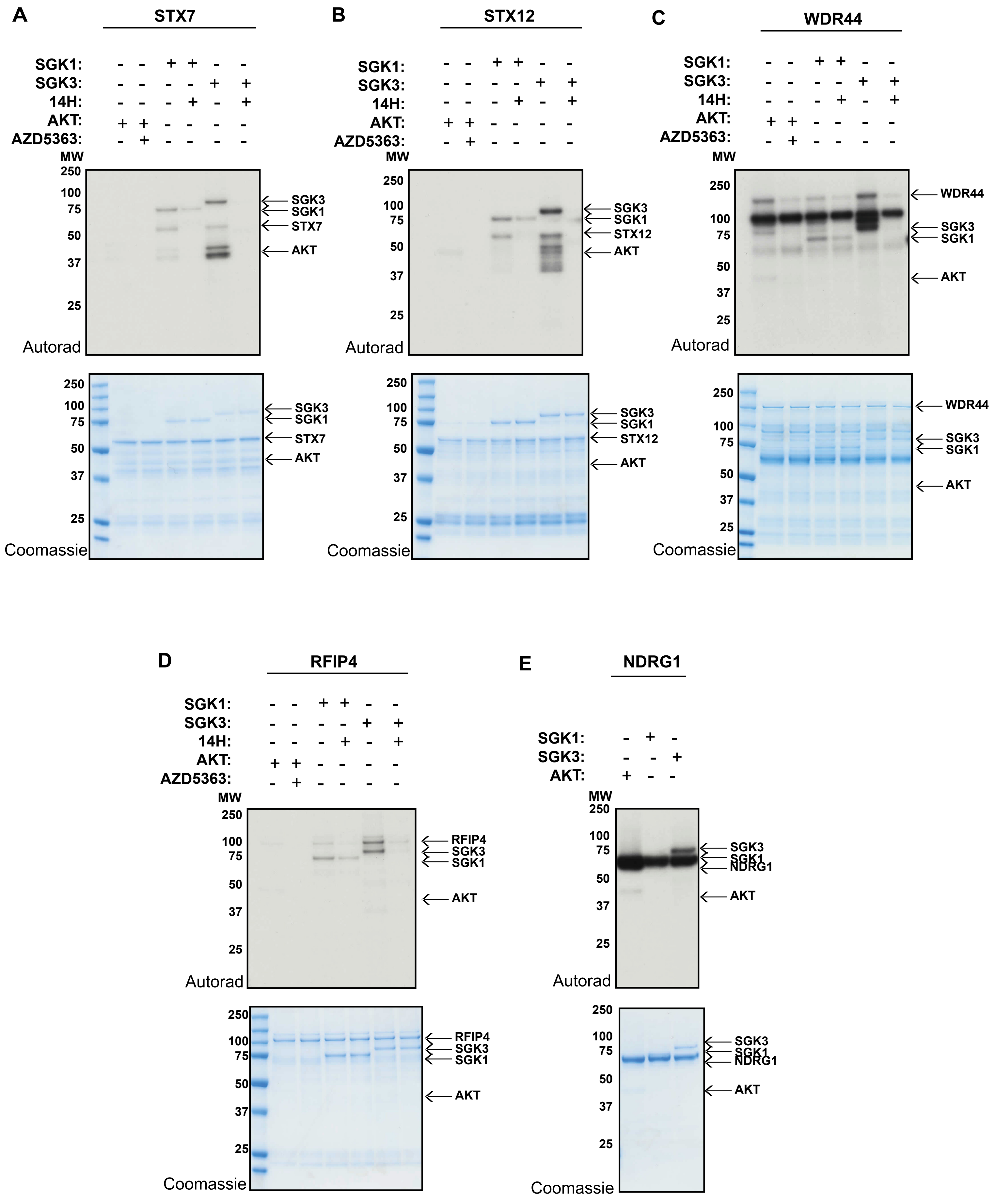
A
STX7
SGK1:
SGK3:
14H:
AKT:
AZD5363:
MW
250
100
75
50
37
25
Autorad
SGK3
SGK1
STX7
AKT
Coomassie
B
STX12
SGK1:
SGK3:
14H:
AKT:
AZD5363:
MW
Autorad
SGK3
SGK1
STX12
AKT
Coomassie
C
WDR44
SGK1:
SGK3:
14H:
AKT:
AZD5363:
MW
Autorad
WDR44
SGK3
SGK1
AKT
Coomassie
D
RFIP4
SGK1:
SGK3:
14H:
AKT:
AZD5363:
MW
Autorad
RFIP4
SGK3
SGK1
AKT
Coomassie
E
NDRG1
SGK1:
SGK3:
AKT:
MW
Autorad
SGK3
SGK1
NDRG1
AKT
Coomassie

Figure S5

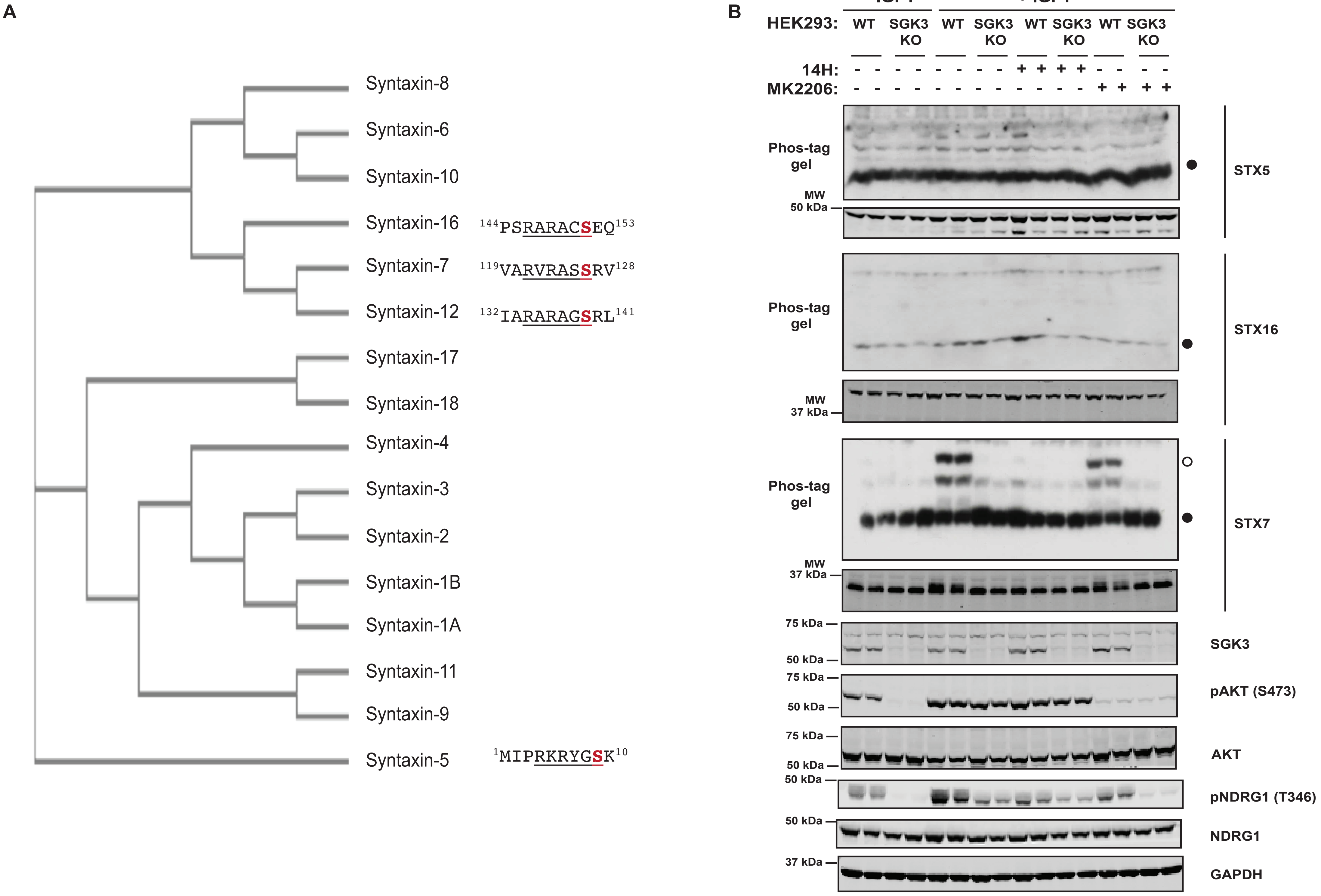
A
Syntaxin-8
Syntaxin-6
Syntaxin-10
Syntaxin-16
144PSRARACSEQ153
Syntaxin-7
119VARVRASSRV128
Syntaxin-12
132IARARAGSRL141
Syntaxin-17
Syntaxin-18
Syntaxin-4
Syntaxin-3
Syntaxin-2
Syntaxin-1B
Syntaxin-1A
Syntaxin-11
Syntaxin-9
Syntaxin-5
1MIPRKRYGSK10
B
- IGF1
+ IGF1
HEK293:
WT
SGK3 KO
14H:
MK2206:
Phos-tag gel
MW
50 kDa
37 kDa
75 kDa
STX5
STX16
STX7
SGK3
pAKT (S473)
AKT
pNDRG1 (T346)
NDRG1
GAPDH

Figure S6

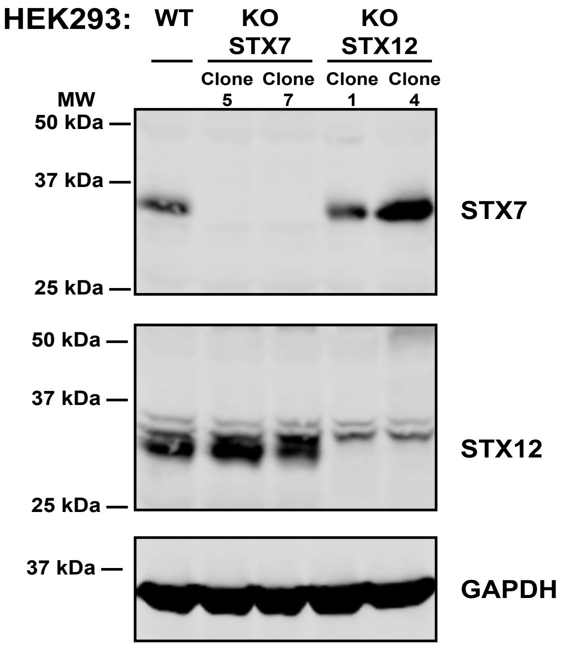
HEK293:
WT
KO
STX7
KO
STX12
Clone 5
Clone 7
Clone 1
Clone 4
MW
50 kDa
37 kDa
25 kDa
STX7
50 kDa
37 kDa
25 kDa
STX12
37 kDa
GAPDH
